# Harnack inequality and one-endedness of UST on reversible random graphs

**DOI:** 10.1007/s00440-023-01239-z

**Published:** 2023-11-08

**Authors:** Nathanaël Berestycki, Diederik van Engelenburg

**Affiliations:** University of Vienna, Vienna, Austria

**Keywords:** Uniform spanning tree, Reversible random graphs, Harnack inequality, Potential theory, Primary 60D05, Secondary 60J45

## Abstract

We prove that for recurrent, reversible graphs, the following conditions are equivalent: (a) existence and uniqueness of the potential kernel, (b) existence and uniqueness of harmonic measure from infinity, (c) a new anchored Harnack inequality, and (d) one-endedness of the wired uniform spanning tree. In particular this gives a proof of the anchored (and in fact also elliptic) Harnack inequality on the UIPT. This also complements and strengthens some results of Benjamini et al. (Ann Probab 29(1):1–65, 2001). Furthermore, we make progress towards a conjecture of Aldous and Lyons by proving that these conditions are fulfilled for strictly subdiffusive recurrent unimodular graphs. Finally, we discuss the behaviour of the random walk conditioned to never return to the origin, which is well defined as a consequence of our results.

## Introduction

### Background and main result

Let (*G*, *o*) be a random unimodular rooted graph, which is almost surely recurrent (with ${\mathbb {E}}(\deg (o)) < \infty$). The **wired Uniform Spanning Tree** (UST for short) on *G* is defined to be the unique weak limit of the uniform spanning tree on any finite exhaustion of the graph, with wired boundary conditions. The existence of this limit is well known, see e.g. [30]. (In fact, since the graph is assumed to be recurrent, the wired or free boundary conditions give the same weak limit). The UST is a priori a spanning forest of the graph *G*, but since *G* is recurrent this spanning forest consists in fact a.s. of a single spanning tree which we denote by $\mathcal {T}$ (see e.g. [33]). We say that $\mathcal {T}$ is **one-ended** if the removal of any finite set of vertices *A* does not disconnect $\mathcal {T}$ into at least two infinite connected components. Intuitively, a one-ended tree consists of a unique semi-infinite path (the spine) to which finite bushes are attached.

The question of the one-endedness of the UST (or the components of the UST, when the graph is not assumed to be recurrent) has been the focus of intense research ever since the seminal work of Benjamini et al. [11]. Among many other results, these authors proved (in Theorem 10.1) that on every unimodular vertex-transitive graph, and more generally on a network with a transitive unimodular automorphism group, every component is a.s. one-ended unless the graph is itself roughly isometric to ${\mathbb {Z}}$ (in which case it and the UST are both two-ended). (This was extended by Lyons et al. [29] to graphs that are neither transitive nor unimodular but satisfy a certain isoperimetric condition slightly stronger than uniform transience). More generally, a conjecture attributed to Aldous and Lyons is that every unimodular one-ended graph is such that every component of the UST is a.s. one-ended. This has been proved in the planar case in the remarkable paper of Angel et al. [1] (Theorem 5.16) and in the transient case by results of Hutchcroft [21, 22]. The conjecture therefore remains open in the recurrent case, which is the focus of this article.

Let us motivate further the question of the one-enededness of the UST. It can in some sense be seen as the analogue^1^ of the question of percolation at the critical value. To see this, note that when the UST is one-ended, every edge can be oriented towards the unique end, so that following the edges forward from any given vertex *w*, we have a unique semi-infinite path starting from *w* obtained by following the edges forward successively. Observe that this forward path necessarily eventually arrives at the spine and moves to infinity along it. Given a vertex *v*, we may define the past ${\textbf {Past}}(v)$ of *v* to be the set of vertices *w* for which the forward path from *w* contains *v*; it is natural to view ${\textbf {Past}}(v)$ as the analogue of a connected component in percolation. From this point of view, the a.s. one-endedness of the tree is equivalent to the finiteness of the past (i.e., connected component in this analogy) of every vertex, as anticipated. We further note that on a unimodular graph, the expected value of the size of the past is however always infinite, as shown by a simple application of the mass transport principle. This confirms the view that the past displays properties expected from a critical percolation model. In fact, Hutchcroft proved in [23] that the two models have same critical exponents in sufficiently high dimension.

In this paper we give necessary and sufficient conditions for the one-endedness of the UST on a recurrent, unimodular graph. These are, respectively: (a) existence of the potential kernel, (b) existence of the harmonic measure from infinity, and finally (c) an anchored Harnack inequality. We illustrate our results by showing that they give straightforward proofs of the aforementioned result of Benjamini, Lyons, Peres and Schramm [11] in the recurrent case (which is one of the most difficult aspects of the proof of the whole theorem, and is in fact stated as Theorem 10.6). We also apply our results to some unimodular random graphs of interest such as the Uniform Infinite Planar Triangulation (UIPT) and related models of infinite planar maps, for which we deduce the Harnack inequality.

To state these results, we first recall the following definitions. Our results can be stated for reversible environments or **reversible random graphs**, i.e., random rooted graphs such that if $X_0$ is the root and $X_1$ the first step of the random walk conditionally given *G* and $X_0$ then $(G, X_0, X_1)$ and $(G,X_1, X_0)$ have the same law. As noted by Benjamini and Curien in [5], any unimodular graph (*G*, *o*) with ${\mathbb {E}}( \deg (o) ) < \infty$ satisfies this reversibility condition after biasing by the degree of *o*. Conversely, any reversible random graph gives rise to a unimodular rooted random graph after unbiasing by the degree of the root. This biasing/unbiasing does not affect any of the results below since they are almost sure properties of the graph. Note also that again by results in [5], a *recurrent* rooted random graph whose law is stationary for random walk is in fact necessarily reversible. See also Hutchcroft and Peres [20] for a nice discussion and Aldous and Lyons [2] for a systematic treatment.

For a nonempty set $A \subset v(G)$ we define the **Green function** by setting for $x \in v(G) \setminus A$ and $y \in v(G)$:

1 $$\begin{aligned} {{\textbf {G}}}_A(x, y) = {\mathbb {E}}_x \left[ \sum _{n = 0}^{T_A - 1} \mathbbm {1}_{\{X_n = y\} }\right] , \end{aligned}$$

where $T_A$ denotes the hitting time of *A*, and $G_A(x, y) = 0$ for $x \in A$. Let

$$\begin{aligned} g_A(x, y):= \frac{{{\textbf {G}}}_A(x, y)}{\deg (y)} \end{aligned}$$

denote the normalised Green function. (Note that due to reversibility, $g_A(x,y) = g_A(y,x)$.)

Let $A_n$ be any (sequence) of finite sets of vertices such that $d(A_n, o) \rightarrow \infty$ as $n \rightarrow \infty$. Here, by $d(A_n, o)$, we just mean the minimal distance of any vertex in $A_n$ to *o*. It is natural to construct the **potential kernel** of the infinite graph *G* by an approximation procedure; we set

2 $$\begin{aligned} a_{A_n}(x, y):= g_{A_n}(y,y) - g_{A_n}(x,y) \end{aligned}$$

In this manner, the potential kernel compares the number of visits to *y*, starting from *x* versus *y*, until hitting the far away set $A_n$. We are interested in existence and uniqueness of limits for $a_{A_n}$ as $n \rightarrow \infty$. In this case we call the unique limit the potential kernel of the graph *G*. We will see that the existence and uniqueness of this potential kernel turns out to be equivalent to a number of very different looking properties of the graph. This definition of the potential kernel differs slightly from the one appearing in [28] for ${\mathbb {Z}}^2$, because we work with a more convenient normalization for graphs that are not transitive. A good example to keep in mind is the graph ${\mathbb {Z}}$ with nearest neighbor edges. Here, the potential kernel is not uniquely defined: for each $m\ge 1$, the sequences $(A_n = \{-n, mn\})_{n\ge 1}$ will give rise to different limits as *m* varies.

We move on to harmonic measure from infinity. Let *A* be a fixed finite, nonempty set of vertices. Let $\mu _n (\cdot )$ denote the harmonic measure on *A*, started from $A_n$ if we wire all the vertices in $A_n$. The **harmonic measure from infinity**, if it exists, is the limit of $\mu _n$ (necessarily a probability measure on *A*).

Now let us turn to Harnack inequality. We say that (*G*, *o*) satisfies an **(anchored) Harnack inequality** (AHI) if there exists an exhaustion $(V_R)_{R \ge 1}$ of the graph (i.e. $V_R$ is a finite subset of vertices and $\cup _{R\ge 1} V_R = v(G)$), and there exists a nonrandom constant $C>0$, such that the following holds. For every function $h: v(G) \rightarrow {\mathbb {R}}_+$ which is harmonic except possibly at 0, and such that $h(0) = 0$:

3 $$\begin{aligned} \max _{x \in \partial V_R} h(x) \le C \min _{x \in \partial V_R} h(x). \end{aligned}$$

The word *anchored* in this definition refers to the fact that the exhaustion is allowed to depend on the choice of root *o*, and the functions are not required to be harmonic there. (As we show in Remark 6.16, a consequence of our results is that an anchored Harnack inequality automatically implies the Elliptic Harnack inequality (EHI) on a suitably defined sequence of growing sets.)

We now state the main theorem.

#### Theorem 1.1

Suppose (*G*, *o*) is a recurrent reversible random graph (or equivalently after unbiasing by the degree of the root, (*G*, *o*) is recurrent unimodular random graph with ${\mathbb {E}}(\deg (o))< \infty$). The following properties are equivalent.

- Almost surely, the pointwise limit of the truncated potential kernel $a_{A_n}(x,y)$ exists and does not depend on the choice of $A_n$.
- Almost surely, the weak limit of the harmonic measure $\mu _n$ from $A_n$ exists and does not depend on $A_n$.
- Almost surely, (*G*, *o*) satisfies an anchored Harnack inequality.
- The uniform spanning tree $\mathcal {T}$ is a.s. one-ended.

Furthermore, if any of these conditions hold, a suitable exhaustion for the anchored Harnack inequality is provided by the sublevel sets of the potential kernel, see Sects. 5 and 6.

### Some applications

**Strengthening of** [11]. Before showing some applications of this result, let us point out that Theorem 1.1 complements and strengthens some of the results of Benjamini, Lyons, Peres and Schramm [11]. In that paper, the (easy) implication (d) implies (b) was noted. We therefore in particular obtain a converse in the reversible case. One can furthermore easily see using their results that on any recurrent planar graph with bounded face degrees (e.g., any recurrent triangulation) (d) holds, i.e., the uniform spanning tree is a.s. one-ended: indeed, for such a graph, there is a rough embedding from the planar dual to the primal, which is assumed to be recurrent, and therefore the planar dual must be recurrent too by Theorem 2.17 in [30]. By Theorem 12.4 in [11] this implies that the uniform spanning tree (on the primal) is a.s. one-ended, and so (d) holds. (In fact, Theorem 5.16 in [1] shows that the bounded face degree assumption is not needed).

**Applications to planar maps.** Therefore, in combination with [11], Theorem 1.1 above applies in particular to unimodular, recurrent triangulations such as the UIPT, or similar maps such as the UIPQ. This therefore implies that these maps have a well-defined potential kernel, harmonic measure from infinity, and satisfy the anchored Harnack inequality. As shown in Remark 6.16, this also implies the **elliptic Harnack inequality** (for sublevel sets of the potential kernel, see Theorem 6.4 for a precise statement). We point out that the elliptic Harnack inequality should not be expected to hold on usual metric balls, but can only be expected on growing sequences of sets which take into account the “natural conformal embedding” of these maps. This is exactly what the potential kernel and its sublevel sets allows us to do.

**More general implications.** We already mention that the equivalence between (a) and (b) is valid more generally, for instance for any locally finite, recurrent graph. The implication (a) $\implies$ (c) to the Harnack inequality (c) is then valid under the additional assumption that the potential kernel grows to infinity (something which we can prove assuming unimodularity). We recall that (d) implies (b) is also true for deterministic graphs, as proved in [11].

#### Remark 1.2

Many of the arguments in this article are true for deterministic graphs. The unimodularity (or reversibility) of the graph with respect to random walk is only used in Lemma 5.4, whose main use is to show that the potential kernel, if it exists, diverges to infinity along any sequence going to infinity (see Lemma 5.5). This property is used for instance in both directions of the relations between (c) and (d), since both go via (a). The unimodularity (or stationarity) is also used to prove that the walks conditioned not to return to the origin satisfy the infinite intersection property, a key aspect of the proof one-endedness. Finally this is also proved to show that if there is a bi-infinite path in the UST then it must essentially be almost space-filling, which is the other main argument of the proof of one-endedness.

**Deterministic case of the Aldous–Lyons conjecture.** As previously mentioned, Theorem 1.1 can be applied to give a direct proof of the one-endedness of the UST for recurrent vertex-transitive graphs not roughly equivalent to ${\mathbb {Z}}$, which is essentially Theorem 10.6 in [11].

#### Corollary 1.3

Suppose *G* is a fixed recurrent, vertex-transitive graph. If *G* is one-ended then the UST is also a.s. one-ended. Otherwise *G* is roughly isometric to ${\mathbb {Z}}$.

#### Proof

Note that *G* must be unimodular, otherwise *G* is nonamenable and so cannot be recurrent (see [38]). Note also that the volume growth of the graph is at most polynomial (as otherwise the walk cannot be recurrent). By results of Trofimov [39], the graph is therefore roughly isometric to a Cayley graph $\Gamma$. Since it is recurrent (as recurrence is preserved under rough isometries, see Theorem 2.17 and Proposition 2.18 of [30]), we deduce by a classical theorem of Varopoulos (see e.g. Theorem 1 and its corollary in [40]) that $\Gamma$ is a finite extension of ${\mathbb {Z}}$ or ${\mathbb {Z}}^2$ and is therefore (as is relatively easily checked) roughly isometric to either of these lattices. Since either of these lattices enjoy the Parabolic Harnack Inequality (PHI), which is, by a consequence of a result proved by Grigoryan [19] and Saloff-Coste [37] independently, preserved under rough isometries (see also [14]), we see that *G* itself satisfies PHI and therefore also the Elliptic Harnack Inequality (EHI): for any $R>1$, if *h* is harmonic in the metric ball *B*(2*R*) of radius 2*R* around the origin, then $\sup _{B(R)} h(x) \le C \inf _{B(R)} h(x)$. (In fact, by a deep recent result of Barlow and Murugan, EHI is now known directly to be stable under rough isometries [12], but here we can appeal to the much simpler stability of PHI. We recommend the following textbooks for related expository material: [25, 4] and [42].)

Suppose that *G* is not roughly isometric to ${\mathbb {Z}}$, therefore it is roughly isometric to ${\mathbb {Z}}^2$. Let us show that *G* satisfies the anchored Harnack inequality (3), with the exhaustion sequence simply obtained by considering metric balls $V_R = B(R)$. Let *h* be nonnegative harmonic on *G* except at 0. Since *G* is rough isometric to ${\mathbb {Z}}^2$, we can cover $\partial V_R$ with a fixed number (say *K*) of balls of radius *R*/10, such that the union of these balls is connected (here we used two-dimensionality). Let $x, y \in \partial V_R$, we can find $x = x_0, \ldots , x_K = y$ with $d (x_i, x_{i+1}) \le R/10$, and $d(x_i, o) > 2R/10$. Exploiting the EHI in each of the *K* balls $B(x_i, 2R/10)$ inductively (since *h* is harmonic in each of these balls), we find that $h(x) \le C^K h(y)$. Since *x*, *y* are arbitrary in $\partial V_R$, this proves the anchored Harnack inequality (3). $\square$

We also show that the one-endedness of the UST holds for unimodular recurrent random graphs if we in addition assume that they are strictly subdiffusive; that is, we settle the Aldous–Lyons conjecture in that case. (This encompasses many models of random planar maps, but can of course hold on more general graphs, see in particular [26], recalled also in Remark 4.2, for sufficient conditions guaranteeing this).

#### Theorem 1.4

Suppose (*G*, *o*) is reversible, almost surely recurrent and strictly subdiffusive (i.e., satisfies (SD) below). Then (*G*, *o*) satisfies (a)–(d).

This applies e.g. for high-dimensional incipient infinite percolation cluster, as explained after Remark 4.2. The proof of Theorem 1.4 takes as an input the results of Benjamini et al. [8] which shows that for strictly subdiffusive unimodular graphs there are no nonconstant harmonic functions of linear growth, and the trivial observation that the effective resistance between points is at most linear in the distance between these points. We believe it should be possible to use the same idea to prove the result assuming only diffusivity: to do this, it would suffice to prove that the effective resistance grows strictly sublinearly, except on graphs roughly isometric to ${\mathbb {Z}}$.

**Random walk conditioned to avoid the origin.** The existence of the potential kernel allows us to define (by *h*-transform) a random walk conditioned to never touch a given point (even though this is of course a degenerate conditioning on recurrent graphs). We study some properties of the conditioned walk and show among other things that two independent conditioned walks must intersect infinitely often, a fact which plays an important role in the proof of Theorem 1.1 for the equivalence between (a) and (d). We conclude the article with a finer study of this conditioned walk on CRT-mated random planar maps. In this case we are able to show that the hitting probability of a point far away from the origin by the conditioned walk remains bounded away from 1 in the limit as the point diverges to infinity (and is bounded away from 0 for “almost all” such points). See Theorem 9.1 for a precise statement. We also discuss a conjecture (see (49)) which, if true, would show a significant difference of behaviours with respect to the more standard case of ${\mathbb {Z}}^2$ (where these hitting probabilities converge to 1/2, as surprisingly shown in [34]).

## Background and notation

Before we begin with the proofs of our theorems, we need to introduce the main notations that we will use throughout this text.

A **graph** *G* consists of a countable collection of vertices *v*(*G*) and edges $e(G) \subset \{\{x, y\}: x, y \in v(G)\}$ and we will always assume that the vertex degrees are finite. We will work with undirected graphs, but will sometimes take the directed edges $\textbf{e}(G) = \{(x, y): \{x, y\} \in e(G)\}$.

The graph *G* comes with a natural metric *d*(*x*, *y*), which is the **graph distance**, i.e. the minimal length of a path between two vertices *x* and *y*. For $n \in {\mathbb {N}}$, we will denote by

$$\begin{aligned} B(y, n) = \{x \in v(G): d(x, y) \le n\}, \end{aligned}$$

the **metric ball** of radius *n*. For a set $A \subset v(G)$, we will write $\partial A$ for its outer boundary in *v*(*G*), that is

$$\begin{aligned} \partial A = \{x \in v(G) \setminus A: \text { there exists a } y \in A \text { with } x \sim y\}. \end{aligned}$$

We will make extensive use of the graph **Laplacian** which we normalise as follows^2^:

4 $$\begin{aligned} \Delta f(x) = \sum _{y \sim x} c(x, y)(f(y) - f(x)), \end{aligned}$$

for functions $f:v(G) \rightarrow {\mathbb {R}}$ (here *c*(*x*, *y*) is the conductance of the edge (*x*, *y*), which is typically equal to one in this paper, except in Sect. 7 where we consider random walk conditioned to avoid the origin forever). A function $h:v(G) \rightarrow {\mathbb {R}}$ is called **harmonic** at *x* if $(\Delta h)(x) = 0$.

Let $X = (X_n)_{n \ge 0}$ denote the simple random walk on *G*, with its law written as ${\mathbb {P}}$ and ${\mathbb {P}}_x$ to mean ${\mathbb {P}}(\cdot \mid X_0 = x)$. For a set $A \subset v(G)$, we define the **hitting time** $T_A = \inf \{n \ge 0: X_n \in A\}$ and $T_x:= T_{\{x\}}$ whenever $A=\{x\}$ consists of just one element. We will write $T_{A}^+$ for the **first return time** to a set *A*. Suppose that *G* is a connected graph. The **effective resistance** is defined through

$$\begin{aligned} {\mathcal {R}}_{\text {eff} }(x \leftrightarrow y):= \frac{{{\textbf {G}}}_x(y, y)}{\deg (y)}. \end{aligned}$$

Recall the useful identity

5 $$\begin{aligned} {\mathcal {R}}_{\text {eff} }(x \leftrightarrow y) = \frac{1}{\deg (y) {\mathbb {P}}_{y}(T_x < T_{y}^+)} \end{aligned}$$

The proof is obvious from the definition of effective resistance when we use the obvious identity

$$\begin{aligned} {{\textbf {G}}}_x(y, y) = \frac{1}{{\mathbb {P}}_y(T_x < T_y^{+})}, \end{aligned}$$

which can be seen by considering the number of excursions from *y* to *y*, which is a geometric random variable by the Markov property.

For infinite graphs *G*, we will say that a sequence of subgraphs $(G_n)_{n \ge 1}$ of *G* is an **exhaustion** of *G* whenever $G_n$ is finite for each *n* and $v(G_n) \rightarrow G$ as $n \rightarrow \infty$. Fix some exhaustion $(G_n)_{n \ge 1}$ of an infinite graph *G* and define the graph $G_n^*$ as $G_n$, together with the identification of $G_n^c$, where we have deleted all self-loops created in the process. For two vertices $x, y \in v(G)$ we recall that

$$\begin{aligned} {\mathcal {R}}_{\text {eff} }(x \leftrightarrow y) = \lim _{n \rightarrow \infty } {\mathcal {R}}_{\text {eff} }(x \leftrightarrow y; G^*_n), \end{aligned}$$

see for instance [30, Section 9.1]. As is well known, the effective resistance defines a metric (see for instance exercise 2.67 in [30]).

Later, we will often work with the metric ${\mathcal {R}}_{\text {eff} }(\cdot \leftrightarrow \cdot )$ on *v*(*G*), instead of the standard graph distance. We introduce the notation

6 $$\begin{aligned} {\mathcal {B}}_{\text {eff} }(x, R) = \{y \in v(G): {\mathcal {R}}_{\text {eff} }(x \leftrightarrow y) \le R\} \end{aligned}$$

for the closed ball with respect to the effective resistance metric. Notice that, in general, this metric space is *not* a length space—making it somewhat inconvenient.

Another result that we will need to use a few times is the ‘last exit decomposition’, or rather two versions thereof which can be proved similarly to [28, Proposition 4.6.4].

### Lemma 2.1

(Last exit decomposition). Let *G* be a graph and $A \subset B \subset v(G)$ finite. Then for all $x \in A$ and $b \in \partial B$ we have

$$\begin{aligned} {\mathbb {P}}_x(X_{T_{B^c}} = b) = \sum _{z \in A} {{\textbf {G}}}_{B^c}(x, z){\mathbb {P}}_z(T_{B^c} < T_A^+, X_{T_{B^c}} = b). \end{aligned}$$

Moreover, for $x \in B$ we have

$$\begin{aligned} {\mathbb {P}}_x(T_A< T_{B^c}) = \sum _{z \in A} {{\textbf {G}}}_{B^c}(x, z){\mathbb {P}}_z(T_{B^c} < T_A^+). \end{aligned}$$

## Equivalence between (a) and (b)

### Base case of equivalence

We will say that a sequence of finite sets of vertices $(A_n)_{n \ge 1}$ ‘goes to infinity’ whenever $d(A_n, o) \rightarrow \infty$ as $n \rightarrow \infty$. Here, by $d(A_n, o)$, we just mean the minimal distance of any vertex in $A_n$ to *o*. Recall the definition of $a_{A_n}$, which also satisfies

7 $$\begin{aligned} a_{A_n}(x, y) = g_{A_n}(y, y) - g_{A_n}(x, y) = \frac{1}{\deg (y)} \frac{{\mathbb {P}}_x (T_{A_n}< T_y)}{ {\mathbb {P}}_y ( T_{A_n} < T_{y}^+) }. \end{aligned}$$

Clearly, both the numerator and the denominator tend to 0 as *n* tends to infinity by recurrence of the underlying graph *G*. When a sequence of subsets $A_n$ has been chosen we will write $a_n$ instead of $a_{A_n}$ with a small abuse of notations.

The goal of this section is to prove the equivalence between (a) and (b) in Theorem 1.1 (in the base case where the set *A* consists of two points; this will be extended to arbitrary finite sets in Sect. 3.3). First, we show that subsequential limits of $a_n$ always exist.

#### Lemma 3.1

Let $(A_n)_{n \ge 1}$ be some sequence of finite sets of vertices going to infinity. There exists a subsequence $(n_k)_{k \ge 1}$ going to infinity such that for all $x, y \in v(G)$ the limit

$$\begin{aligned} a(x,y): = \lim _{k \rightarrow \infty } a_{{n_k}}(x, y) \end{aligned}$$

exists in $[0, \infty )$. Moreover, $a(x,y) > 0$ precisely when the removal of *y* from *G* does not disconnect *x* from $A_{n_k}$ for all *k* large enough.

#### Proof

Fix $y \in v(G)$ and suppose first that for all $u \sim y$ we have ${\mathbb {P}}_u(T_{A_n} < T_y) > 0$ for all *n* large enough (i.e., *y* does not disconnect a portion of the graph from infinity).

Let $x \in v(G)$ and fix *n* so large that $A_n$ does not contain *y*, *x* or any of the neighbors of *y*. For each $u \sim y$, we can force the random walk started from *x* to go through *u* before touching $A_n$ or *y* to get

8 $$\begin{aligned} {\mathbb {P}}_x(T_{A_n}< T_y) \ge {\mathbb {P}}_x(T_u< T_{A_n} \wedge T_y){\mathbb {P}}_u(T_{A_n} < T_y). \end{aligned}$$

Upon taking $u \sim y$ such that it maximizes ${\mathbb {P}}_u(T_{A_n} < T_y)$ and by recurrence of *G* we get the existence of $c(x, y) > 0$ for which

$$\begin{aligned} a_{n}(x, y) = \frac{{\mathbb {P}}_x(T_{A_n}< T_y)}{\sum _{u \sim y} {\mathbb {P}}_u(T_{A_n}< T_y)} \ge \frac{{\mathbb {P}}_x(T_u < T_{A_n} \wedge T_y)}{\deg (y)} \ge c(x, y) >0. \end{aligned}$$

The same reasoning as in (8) but in the other direction gives

$$\begin{aligned} {\mathbb {P}}_x(T_{A_n}< T_y) \le \frac{{\mathbb {P}}_u(T_{A_n}< T_y)}{{\mathbb {P}}_u(T_x < T_{A_n} \wedge T_y)}. \end{aligned}$$

Hence, using again recurrence of *G* we get that there is some $C(x, y) < \infty$ such that (upon taking the right *u*)

$$\begin{aligned} a_{n}(x, y) \le \frac{{\mathbb {P}}_u(T_{A_n}< T_y)}{{\mathbb {P}}_u(T_x< T_{A_n} \wedge T_y)\sum _{u \sim y}{\mathbb {P}}_u(T_{A_n}< T_y)} \le C(x, y) < \infty . \end{aligned}$$

We deduce that for fixed *x*, *y*, subsequential limits of $a_{n}(x, y)$ exist and the existence of subsequential limits for all *x*, *y* simultaneously follows from diagonal extraction.

The existence of subsequential limits in the general case is the same as we can always lower bound $a_n(x, y)$ by 0 and the upper bound does not change.

Now, if $x \in v(G)$ is such that the removal of *y* disconnects *x* from $A_{n_k}$, then $a_{n_k}(x, y) = 0$. Suppose thus that *x* is such that the removal of *y* does not disconnect *x* from $A_{n_k}$ for all *k* large enough. In this case, we can restrict ourselves to just the component of *G* with *y* removed, in which both $A_{n_k}$ and *x* are as the hitting probabilities are the same in this case. Hence, we are back in the situation above and $a_{n_k}(x, y) \ge c(x, y) > 0$. $\square$

We next present a result, which shows that any subsequential limit appearing in Lemma 3.1 must satisfy a certain number of properties.

#### Proposition 3.2

Let *a*(*x*, *y*) be any subsequential limit as in Lemma 3.1. Then $a:v(G) \rightarrow {\mathbb {R}}_+$ satisfies

(i) for each $$y \in v(G)$$
  $$\begin{aligned} \Delta a(\cdot , y) = \delta _y(\cdot ) \qquad \text { and } \qquad a(y, y) = 0, \end{aligned}$$
  where we recall that $\Delta$ is defined in (4) and is normalised so that $\Delta f(x) = \sum _y (f(y) - f(x))$.
(ii) for all $x, y \in v(G)$ we have
  $$\begin{aligned} a(x, y) = \lim _{k \rightarrow \infty }{\mathbb {P}}_{A_{n_k}}(T_x < T_y){\mathcal {R}}_{\text {eff} }(x \leftrightarrow y), \end{aligned}$$
  where ${\mathbb {P}}_A$ refers to the law of a random walk starting from *A*, when all of the vertices in *A* have been wired together.

The equivalence between (a) and (b) of Theorem 1.1 (in the base case where the finite set *B* on which we need to define harmonic measure consists of two points) is then obvious, and we collect it here:

#### Corollary 3.3

Let *G* be a recurrent graph. Then

$$\begin{aligned} {{\text {hm}}}_{x, y}(x):= \lim _{n \rightarrow \infty } {\mathbb {P}}_{A_n}(T_x < T_y) \end{aligned}$$

exists for all $x, y \in v(G)$ and is independent of the sequence $(A_n)_n$ if and only if the potential kernel is uniquely defined. Furthermore, in this case,

$$\begin{aligned} a(x, y) = {{\text {hm}}}_{x, y}(x){\mathcal {R}}_{\text {eff} }(x \leftrightarrow y). \end{aligned}$$

#### Proof of Proposition 3.2

The proof of item (i) is rather elementary. Fix $y \in v(G)$ and $n \ge 1$. Since $x \mapsto {\mathbb {P}}_x(T_{A_{n}} < T_y)$ is a harmonic function outside of *y* and $A_{n}$ by the simple Markov property, we get that $x \mapsto a_{n}(x, y)$ is harmonic outside *y* and $A_{n}$, see (7). It follows that $x \mapsto a(x, y)$ is harmonic at least away from *y*. Furthermore, note that $a_{n}(y, y) = 0$ by definition and

$$\begin{aligned} \sum _{u \sim y} a_{n}(u, y) = \frac{\sum _{u \sim y} {\mathbb {P}}_u(T_{A_{n}}< T_y)}{\sum _{u \sim y} {\mathbb {P}}_u(T_{A_{n}} < T_y)} = 1 \end{aligned}$$

so $\Delta a_n( \cdot , y )|_{\cdot =y } = 1$. This finishes the proof of (i).

For part (ii), we notice first that by properties of the electrical resistance,

$$\begin{aligned} \sum _{u \sim y} {\mathbb {P}}_u(T_{A_{n}}< T_y) = \deg (y){\mathbb {P}}_y( T_{A_{n}} < T_y^+) = \frac{1}{{\mathcal {R}}_{\text {eff} }(y \leftrightarrow A_{n})}, \end{aligned}$$

which allows us to write

9 $$\begin{aligned} a_{n}(x, y) = {\mathcal {R}}_{\text {eff} }(y \leftrightarrow A_{n}) {\mathbb {P}}_x(T_{A_{n}} < T_y). \end{aligned}$$

Identify the vertices in $A_{n}$ and delete possible self-loops created in the process. The resulting graph $G'_{n}$ is then still recurrent. Let ${{\textbf {G}}}_y(\cdot , \cdot )$ denote the Green function on this graph when the walk is killed at *y*. We can also express the effective resistance in terms of the normalised Green function: that is,

$$\begin{aligned} {\mathcal {R}}_{\text {eff} }(y \leftrightarrow A_n) = \frac{{{\textbf {G}}}_y (A_n, A_n)}{\deg (A_n)} \end{aligned}$$

Using the Markov property and since $G'_n$ is reversible,

10 $$\begin{aligned} a_{n}(x, y)&= {\mathbb {P}}_x(T_{A_n}< T_y) \frac{{{\textbf {G}}}_y(A_{n}, A_{n})}{\deg (A_{n})} \nonumber \\&= \frac{{{\textbf {G}}}_{y}(x, A_{n})}{\deg (A_{n})}\nonumber \\&= \frac{{{\textbf {G}}}_y (A_n, x)}{\deg (x)}\\&= {\mathbb {P}}_{A_{n}}(T_x < T_y){\mathcal {R}}_{\text {eff} }(x \leftrightarrow y; G'_n)\nonumber \end{aligned}$$

by using the same argument in the other direction, and where the effective resistance in the last line is calculated in $G'_n$.

Since the graph *G* is recurrent, it follows that ${\mathcal {R}}_{\text {eff} }(x \leftrightarrow y; G'_n)$ converges to ${\mathcal {R}}_{\text {eff} }(x \leftrightarrow y; G)$ as $n \rightarrow \infty$ (as the free and wired effective resistances agree). We deduce that

$$\begin{aligned} a(x, y) = \lim _{k \rightarrow \infty } {\mathbb {P}}_{A_{n_k}}(T_x < T_y){\mathcal {R}}_{\text {eff} }(x \leftrightarrow y), \end{aligned}$$

which finishes part (ii). $\square$

#### Remark 3.4

We wish to point out that, in general, the potential kernels are *not* symmetric (even if they are uniquely defined).

### Triangle inequality for the potential kernel

Before we start of the proof of the remaining implications, we need some preliminary estimates on the potential kernel, showing that it satisfies a form of triangle inequality. This plays a crucial role throughout the rest of this paper. We also need a decomposition of the potential kernel in order to prove that for reversible graphs, the potential kernel (if it is well defined) satisfies the growth condition.

We start with a simple and well known application of the optional stopping theorem:

#### Lemma 3.5

Let *A* be some finite set and suppose that $x, y \in A$. Then

$$\begin{aligned} \frac{{{\textbf {G}}}_{A^c}(x, y)}{\deg (y)} = {\mathbb {E}}_x[a(X_{T_{A^c}}, y)] - a(x, y). \end{aligned}$$

#### Proof

This is Proposition 4.6.2 in [28], but we include for completeness since its proof if simple. Let $x, y \in A$ and notice that

$$\begin{aligned} M_n:= a(X_n, y) - \sum _{j=0}^{n - 1} \frac{\delta _y(X_j)}{\deg (y)} \end{aligned}$$

is a martingale. Applying the optional stopping theorem at $T_{A^c} \wedge n$, we obtain

$$\begin{aligned} a(x, y) = {\mathbb {E}}_{x}[M_0] = {\mathbb {E}}_x[a(X_{n \wedge T_{A^c}}, y)] - \frac{1}{\deg (y)}{\mathbb {E}}_x \left[ \sum _{j=0}^{(n \wedge T_{A^c}) - 1} \delta _y({X_j })\right] , \end{aligned}$$

Taking $n \rightarrow \infty$, since *A* is finite, we deduce from dominated (resp. monotone) convergence that

$$\begin{aligned} {\mathbb {E}}_x[a(X_{n \wedge T_{A^c}}, y)] \rightarrow {\mathbb {E}}_x[a(X_{T_{A^c}}, y)], \frac{1}{\deg (y)}{\mathbb {E}}_x \left[ \sum _{j=0}^{(n \wedge T_{A^c}) - 1} \delta _y(X_j)\right] \rightarrow \frac{{{\textbf {G}}}_{A^c}(x, y)}{\deg (y)}, \end{aligned}$$

showing the result. $\square$

#### Proposition 3.6

Let $x, y, z \in v(G)$ be three vertices. We have the identity

$$\begin{aligned} \frac{{{\textbf {G}}}_z(x, y)}{\deg (y)} = a(x, z) - a(x, y) + a(z, y). \end{aligned}$$

#### Proof

Fix $x, y, z \in v(G)$ and let $(A_n)_{n \ge 1}$ be some sequence of finite sets of vertices going to infinity^3^.

Glue together $A_n$ on the one hand, and the vertices of $B(o, m)^c$ on the other hand. Delete all self-loops created in the process and write $\partial _m$ for the vertex corresponding to $B(o, m)^c$. Let ${\tilde{X}}_k$ be the simple random walk on the graph obtained from gluing $A_n$ and $\partial _m$. We define for $w, w' \in B(o, m) \cup \{\partial _m\}$ the function

$$\begin{aligned} a_{m, n}(w, w'):= {\mathcal {R}}_{\text {eff} }(\{\partial _m, w'\} \leftrightarrow A_n){\mathbb {P}}_{w}(T_{A_n} < T_{w'} \wedge T_{\partial _m}). \end{aligned}$$

By recurrence and (9), we have that $a_{m, n}(w, w') \rightarrow a_n(w, w')$ as $m \rightarrow \infty$, for all $w, w'$.

Fix *n* so large that *x*, *y* and *z* are not in $A_n$. Let *m* be so large that *x*, *y*, *z* and $A_n$ are in *B*(*o*, *m*). Define $E_{n, m} = \{A_n, z, \partial _m\}$. Then, as in Lemma 3.5,

11 $$\begin{aligned} a_{m, n}(x, y) = {\mathbb {E}}_x[a_{m, n}({\tilde{X}}_{T_{E_{m, n}}}, y)] - \frac{{{\textbf {G}}}_{E_{m,n}}(x,y)}{\deg (y)} \end{aligned}$$

On the other hand, by definition of $E_{m,n}$ we have

$$\begin{aligned} {\mathbb {E}}_{x}[a_{m, n}(X_{T_{E_{m, n}}}, y)]&= {\mathbb {P}}_x(T_z< T_{A_n} \wedge T_{\partial _m})a_{m, n}(z, y) \\&\quad + {\mathbb {P}}_x(T_{A_n}< T_z \wedge T_{\partial _m})a_{m, n}(A_n, y) \\&\quad + {\mathbb {P}}_x(T_{\partial _m} < T_{A_n} \wedge T_z)a_{m, n}(\partial _m, y), \end{aligned}$$

where a priori the hitting probabilities are calculated on the graph where $A_n$ and $\partial _m$ are glued. However, as we are only interested in the first hitting time of either of these sets, it does not matter and we can calculate the probabilities also for the random walk on the graph *G*. Notice that, by definition, $a_{m, n}(\partial _m, y) = 0$. Plugging this back into (11) we obtain

$$\begin{aligned} a_{m, n}(x, y)= & {} {\mathbb {P}}_x(T_z< T_{A_n} \wedge T_{\partial _m})a_{m, n}(z, y) + {\mathbb {P}}_x(T_{A_n} < T_z \wedge T_{\partial _m})a_{m, n}(A_n, y) \\{} & {} \quad - \frac{{{\textbf {G}}}_{E_{m, n}}(x, y)}{\deg (y)}. \end{aligned}$$

We have already observed that $a_{m, n}(w, y) \rightarrow a_n(w, y)$ for each *w* as $m \rightarrow \infty$. Then, by recurrence of *G* and monotone convergence, we get

12 $$\begin{aligned} a_n(x, y) = {\mathbb {P}}_x(T_z< T_{A_n})a_{n}(z, y) + {\mathbb {P}}_x(T_{A_n} < T_z)a_{n}(A_n, y) - \frac{{{\textbf {G}}}_{\{A_n, z\}}(x, y)}{\deg (y)}. \nonumber \\ \end{aligned}$$

Next, we wish to take $n \rightarrow \infty$. The left-hand side converges to *a*(*x*, *y*) as $n \rightarrow \infty$, by definition of the potential kernel. The first term on the right-hand side converges to *a*(*z*, *y*) by the same argument and recurrence of the graph *G*. Using once more monotone convergence, we find

13 $$\begin{aligned} \frac{{{\textbf {G}}}_{\{A_n, z\}}(x, y)}{\deg (y)} \rightarrow \frac{{{\textbf {G}}}_z(x, y)}{\deg (y)} \end{aligned}$$

as *n* goes to infinity. We are left to deal with the term ${\mathbb {P}}_x(T_{A_n} < T_z)a_{n}(A_n, y)$, which we claim converges to *a*(*x*, *z*).

From the definition of $a_n$, together with the representation in (9), we find

$$\begin{aligned} a_{n}(A_n, y) = {\mathcal {R}}_{\text {eff} }(y \leftrightarrow A_n){\mathbb {P}}_{A_n}(T_{A_n} < T_y) = {\mathcal {R}}_{\text {eff} }(y \leftrightarrow A_n). \end{aligned}$$

Thus, using again the same representation of $a_n(x, z)$, we see that

$$\begin{aligned} a_n(A_n, y) {\mathbb {P}}_x(T_{A_n}< T_z)&= {\mathcal {R}}_{\text {eff} }(y \leftrightarrow A_n){\mathbb {P}}_x(T_{A_n} < T_z) \\&= a_{n}(x, z)\frac{{\mathcal {R}}_{\text {eff} }(y \leftrightarrow A_n)}{{\mathcal {R}}_{\text {eff} }(z \leftrightarrow A_n)}. \end{aligned}$$

Using the triangle inequality for the effective resistance, we notice that

$$\begin{aligned} \frac{{\mathcal {R}}_{\text {eff} }(y \leftrightarrow A_n)}{{\mathcal {R}}_{\text {eff} }(z \leftrightarrow y) + {\mathcal {R}}_{\text {eff} }(y \leftrightarrow A_n)} \le \frac{{\mathcal {R}}_{\text {eff} }(y \leftrightarrow A_n)}{{\mathcal {R}}_{\text {eff} }(z \leftrightarrow A_n)} \le \frac{{\mathcal {R}}_{\text {eff} }(y \leftrightarrow z) + {\mathcal {R}}_{\text {eff} }(z \leftrightarrow A_n)}{{\mathcal {R}}_{\text {eff} }(z \leftrightarrow A_n)}. \end{aligned}$$

By recurrence of *G*, the left and right hand side converge to 1 as $n \rightarrow \infty$. In particular, we deduce that

$$\begin{aligned} a_n(A_n, y){\mathbb {P}}_x(T_{A_n} < T_z) \rightarrow a(x, z) \end{aligned}$$

as $n \rightarrow \infty$. Plugging this, together with (13) back into (12) we conclude:

$$\begin{aligned} a(x, y) = a(x, z) + a(z, y) - \frac{{{\textbf {G}}}_z(x, y)}{\deg (y)} \end{aligned}$$

as desired. $$\square $$

#### Remark 3.7

Proposition 3.6 is an extensions of results known for the lattice ${\mathbb {Z}}^2$, see Proposition 4.6.3 in [28] and the discussion thereafter. As far as we know, these proofs are based on precise asymptotic behavior of the potential kernel, a tool we do not seem to have.

#### Remark 3.8

The statement of Proposition 3.6 is also valid for an arbitrary subsequential limit $a(\cdot , \cdot )$ of $a_n (\cdot , \cdot )$, even when a proper limit is not known to exist. In particular, it shows that given such a subsequential limit $a( \cdot , y)$ there is a unique way to coherently define $a(\cdot , z)$. For this reason, if $\lim _{n \rightarrow \infty } a_n(x, y)$ is shown to exist for a fixed *y* and *all* $x \in v(G)$, it follows that this limit exists for *all* $x, y \in v(G)$ simultaneously. This will be used in Theorem 3.11.

#### Corollary 3.9

For each $x, z \in v(G)$ and all $\epsilon > 0$ there exists an $N = N(\epsilon , x, z)$ such that for all *y* with $d(x, y) \ge N$ we have

$$\begin{aligned} |a(x, y) - a(z, y)| \le \epsilon \end{aligned}$$

and in particular $\lim _{n \rightarrow \infty } a(x, y_n) - a(z, y_n) = 0$ for any sequence $(y_n)_{n \ge 1}$ going to infinity.

Notice that Corollary 3.9 does *not* say that $a(y_n, x) - a(y_n, z) \rightarrow 0$ as $n \rightarrow \infty$ in general! Indeed, a similar argument shows that $a(y_n, x) - a(y_n, z) \rightarrow a(z, x) - a(x, z)$, which is nonzero in general.

#### Proof

Fix $x, z \in v(G)$ and suppose by contradiction that there is some $\epsilon > 0$, such that for infinitely many $n \ge 1$ (but in fact we can with a small abuse of notation assume for all $n\ge 1$ after taking a subsequence), there is some $y_n$ with $d(x, y_n) \ge n$ for which

$$\begin{aligned} |a(z, y_n) - a(x, y_n)| > \epsilon . \end{aligned}$$

By Proposition 3.6 and $\deg (\cdot )$-reversibility of the Simple Random Walk we have

$$\begin{aligned} a(x, y_n) - a(z,y_n) = a(x, z) - \frac{{{\textbf {G}}}_z (x, y_n)}{\deg (y_n)} = a(x,z) - \frac{{{\textbf {G}}}_z (y_n,x)}{\deg (x)}. \end{aligned}$$

Take $A_n = \{y_n\}$ and recall (see e.g. (10)) that

$$\begin{aligned} a_n(x, z) = \frac{{{\textbf {G}}}_z(y_n, x)}{\deg (x)}. \end{aligned}$$

Therefore

$$\begin{aligned} a(x, y_n) - a(z, y_n) = a(x,z) - a_n(x, z). \end{aligned}$$

Since this converges to zero as $n \rightarrow \infty$, we get the desired contradiction. $\square$

We immediately deduce that the harmonic measures from infinity of $\{x, y\}$ and $\{z, y\}$ are very similar if *y* is far away from *x* and *z*.

#### Corollary 3.10

Fix $x, z \in v(G)$. For every $\epsilon > 0$, there exists an $N = N(x, z, \epsilon )$ such that for all *y* with $d(x, y) \ge N$ we have

$$\begin{aligned} |{{\text {hm}}}_{x, y}(x) - {{\text {hm}}}_{z,y}(z)| \le \frac{\epsilon + {\mathcal {R}}_{\text {eff} }(z \leftrightarrow x)}{{\mathcal {R}}_{\text {eff} }(x \leftrightarrow y)}. \end{aligned}$$

#### Proof

Fix $x, z \in v(G)$ and $\epsilon > 0$. Let $N_0$ be so large that Corollary 3.9 holds, i.e. so that for every *y* with $d(x, y) \ge N_0$,

$$\begin{aligned} |a(x, y) - a(x, z)| \le \epsilon . \end{aligned}$$

Recall from Corollary 3.3 the expression

$$\begin{aligned} a(x, y) = {{\text {hm}}}_{x, y}(x) {\mathcal {R}}_{\text {eff} }(x \leftrightarrow y). \end{aligned}$$

so that

$$\begin{aligned} {{\text {hm}}}_{x, y}(x) - {{\text {hm}}}_{z, y}(z) = \frac{a(x, y) - a(z, y)}{{\mathcal {R}}_{\text {eff} }(x \leftrightarrow y)} + \frac{{{\text {hm}}}_{z, y}(z)({\mathcal {R}}_{\text {eff} }(x \leftrightarrow y) - {\mathcal {R}}_{\text {eff} }(z \leftrightarrow y))}{{\mathcal {R}}_{\text {eff} }(x \leftrightarrow y)}. \end{aligned}$$

Last, using the triangle inequality for the effective resistance twice (and symmetry ${\mathcal {R}}_{\text {eff} }(x \leftrightarrow y) = {\mathcal {R}}_{\text {eff} }(y \leftrightarrow x)$), we find

$$\begin{aligned} |{\mathcal {R}}_{\text {eff} }(x \leftrightarrow y) - {\mathcal {R}}_{\text {eff} }(z \leftrightarrow y)| \le {\mathcal {R}}_{\text {eff} }(x \leftrightarrow z). \end{aligned}$$

Plugging this all together and defining $N \ge N_0$ so large that ${\mathcal {R}}_{\text {eff} }(x \leftrightarrow y) \ge \frac{1}{\epsilon }{\mathcal {R}}_{\text {eff} }(x \leftrightarrow z)$ for all *y* with $d(x, y) \ge N$, gives that

$$\begin{aligned} |{{\text {hm}}}_{x, y}(x) - {{\text {hm}}}_{z, y}(z)| \le \epsilon + \epsilon , \end{aligned}$$

which is the desired result. $\square$

### Gluing and harmonic measure

We suppose throughout this section that the potential kernel is well defined in the sense that the subsequential limits appearing in Lemma 3.1 are all equal. By Corollary 3.3, this implies that the harmonic measure from infinity is well defined for two-point sets.

Let $B \subset v(G)$ be a set. Glue together all vertices in *B* and delete all self-loops that were created in the process. We denote the graph induced by the gluing $G_B$. Note that $G_B$ need not be a simple graph, even when *G* was.

We will prove in this section that, if the potential kernel is well defined on *G*, it is also well defined on $G_B$, whenever *B* is a finite set. Furthermore, we will prove an explicit expression of the potential kernel on the graph $G_B$ in the case where *B* is a finite set. These results are an extension of results on the lattice ${\mathbb {Z}}^2$, see for instance [28, Chapter 6], but we will use different arguments, following from the expression for the potential kernel in terms of harmonic measure from infinity as in Corollary 3.3.

#### Theorem 3.11

(Gluing Theorem) Suppose $$a(x,y) = \lim _{n \rightarrow \infty } a_n(x,y)$$ exists for all $$x,y \in v(G)$$ and does not depend on the choice of the sequence of sets $$A_n $$ going to infinity. Let $$B \subset v(G)$$ be a finite set, whose removal does not disconnect *G*, and suppose $$x \in B$$. Then

14 $$\begin{aligned} q_B(w):= \lim _{n\rightarrow \infty } {\mathcal {R}}_{\text {eff} }(B\leftrightarrow A_n){\mathbb {P}}_w( T_{A_n} < T_B) \end{aligned}$$

exists and is given by

15 $$\begin{aligned} q_B(w) = a(w, x) - {\mathbb {E}}_w[a(X_{T_B}, x)]; \ \ w \in v(G_B) \setminus \{B\}; q_B(B) =0. \end{aligned}$$

Extending $q_B$ to *v*(*G*) in the natural way (i.e., using (15) with $w \in v(G)$), we have

16 $$\begin{aligned} (\Delta q_B)(w) = {{\text {hm}}}_B(w):= \lim _{z \rightarrow \infty } {\mathbb {P}}_z(X_{T_B} = w); \ \ w \in B \end{aligned}$$

where the Laplacian $\Delta$ is calculated on *G* via (4).

Note in particular, that in the expression (15) for $q_B$, any choice of $x \in B$ gives the same value and so is irrelevant. We will prove this theorem in the two subsequent subsections, proving first (14) and (15) in Sect. 3.3.1, and then (16) in Sect. 3.3.2.

Before we give the proof, we first state some corollaries. The first one is that the harmonic measure from infinity is well defined for the arbitrary finite set *B* (subject to the assumption that the removal of *B* does not disconnect *G*).

#### Corollary 3.12

Fix a finite set $B \subset v(G)$ as in Theorem 3.11. Let $A_n$ be a set of vertices tending to infinity. Then for any $x \in B$,

17 $$\begin{aligned} {{\text {hm}}}_B (x) = \lim _{n \rightarrow \infty } {\mathbb {P}}_{A_n} (X_{T_B} = x) \end{aligned}$$

exists and is positive for all $x \in B$ such that the removal of $B {\setminus } \{x\}$ does not disconnect *x* from infinity.

#### Proof

Fix $w \notin B$, then arguing as in (9) and (10) we get

$$\begin{aligned} {\mathbb {P}}_{A_n} (T_w< T_B ) = \frac{ {\mathcal {R}}_{\text {eff} }(A_n \leftrightarrow B) {\mathbb {P}}_w ( T_{A_n} < T_B)}{ {\mathcal {R}}_{\text {eff} }(w \leftrightarrow B; G_{A_n})} \rightarrow \frac{q_B(w) }{{\mathcal {R}}_{\text {eff} }(w \leftrightarrow B)} \end{aligned}$$

as $n \rightarrow \infty$. This limit is by definition the desired value of ${{\text {hm}}}_{B \cup \{w\}} (w)$. Note furthermore that $q_B(w)$ is strictly positive by Lemma 3.1.

Applying the same reasoning but with *B* changed into $B' = B {\setminus }\{x\}$ (with $x \in B$) and $w =x$, shows that the limit in (17) exists. Furthermore, if the removal of $B'$ does not disconnect *x* from $\infty$, we see that $q_{B'}(w) >0$ again, and so ${{\text {hm}}}_B(x) >0$. $\square$

Next, we show that the potential kernel can only be well defined if the graph *G* is one-ended.

#### Corollary 3.13

If the potential kernel is well defined, *G* is one-ended.

#### Proof

Intuitively, on multiple-ended graphs there isn’t a single harmonic measure from infinity since there are several ways of converging to infinity. Suppose *G* has more than one end. Let $x_1, x_2, \ldots , x_M$ be some finite number of vertices, such that removing them from *v*(*G*) and looking at the induced graph, we have (at least) two infinite components. Write $B_n = B(o, n)$ and choose *n* large enough that $x_1, \ldots , x_M \in B_n$. Consider the graph $G_{B_n}$ resulting from gluing $B_n$ together as in the theorem. Clearly, the removal of $B_n$ creates at least two infinite components. Pick a vertex *z* of $B_n^c$ and suppose it is in one infinite component. Let $(\{w_{i}\})_{i \ge 1}$ be any sequence of vertices going to infinity in an infinite component that does not contain *z*. Then ${\mathbb {P}}_{w_i}(T_{z} < T_{B_n}) = 0$ (for each *i*), yet this converges by Corollary 3.12 to ${{\text {hm}}}_{B_n \cup {z}} (z)>0$ since the removal of $B_n$ does not disconnect *z* from infinity. This is the desired contradiction. $\square$

Theorem 3.11 a priori only shows that the potential kernel with ‘pole’ *B* is well defined when *B* does not disconnect *G*. We can, however, extend it to arbitrary finite sets *B* and to an arbitrary second variable *y*.

#### Corollary 3.14

Let $B \subset v(G)$ be *any* finite set. The potential kernel $a_B:v(G_B)^2 \rightarrow {\mathbb {R}}_+$ is well defined in the sense that the limit

$$\begin{aligned} a^{G_B}(w, y) = \lim _{n\rightarrow \infty } {\mathbb {P}}_w(T_{A_n} < T_y; G_B){\mathcal {R}}_{\text {eff} }(w \leftrightarrow y; G_B), \end{aligned}$$

exist for all $w, y \in v(G_B)$ and does not depend on the choice of sequence of sets $A_n$. Here, the probability and effective resistance are calculated on the graph $G_B$.

#### Proof

We start with taking ${\bar{B}}$ as the hull (in the sense of complex analysis, meaning we “fill it in” with respect to the point at infinity) of *B*, defined by adding to *B* all the points in $v(G_B)$ that belong to *finite* connected components of $v(G_B) \setminus B$. Since *G* is one-ended by Corollary 3.13, ${\bar{B}}$ does not disconnect *G*. By Theorem 3.11, we have that for any sequence of sets $(A_n)_{n \ge 1}$ going to infinity, the limit

$$\begin{aligned} a^{G_{{\bar{B}}}}(w, {\bar{B}}):= q_{{\bar{B}}}(w) = \lim _{n \rightarrow \infty } {\mathbb {P}}_{w}(T_{A_n} < T_{{\bar{B}}}){\mathcal {R}}_{\text {eff} }( {\bar{B}} \leftrightarrow A_n) \end{aligned}$$

exists for each $w \in v(G_{{\bar{B}}})$ and does not depend on the choice of sequence of vertices $A_n$ going to infinity. Moreover, this limit also trivially exists (and is zero) if *w* is in one of the finite components of $v(G) \setminus B$.

Hence we deduce that actually for all $w \in v(G_B)$ we have that the limit

$$\begin{aligned} a^{G_B}(w, B) = \lim _{n\rightarrow \infty } {\mathbb {P}}_w(T_{A_n} < T_B){\mathcal {R}}_{\text {eff} }(A_n \leftrightarrow B) \end{aligned}$$

exists and does not depend on the choice of sequence of sets going to infinity $A_n$. Now, by Proposition 3.6 (see Remark 3.8) we get that for *all* $w, y \in v(G_B)$ the limit

$$\begin{aligned} a^{G_B}(w, y) = \lim _{n\rightarrow \infty } {\mathbb {P}}_w(T_{A_n} < T_y; G_B){\mathcal {R}}_{\text {eff} }(A_n \leftrightarrow y; G_B) \end{aligned}$$

exists and does not depend on the choice of the sequence $A_n$. This is the desired result. $\square$

#### Proof of (14) and (15)

##### Proof

Fix $(A_n)_{n \ge 1}$ a sequence of finite sets of vertices going to infinity. For a finite set $B \subset v(G)$ and $x \in B$, we will define the function $q_B:v(G_B) \rightarrow {\mathbb {R}}_+$ through

$$\begin{aligned} q_B(w) = a(w, x) - {\mathbb {E}}_w[a(X_{T_B}, x)], \end{aligned}$$

and $q_B(B) = 0$, whenever the potential kernel on *G* is well defined. We will prove (14) using induction on the number of vertices *m* in *B*. To be more precise, we will show that for any recurrent graph *G* for which the potential kernel is well defined (in other words, $\lim _{n\rightarrow \infty } a_{A_n}(x, y) = a(x, y)$ and does not depend on the sequence $A_n$) for any set $B \subset v(G)$ with $|B| = m$ and $v(G) \setminus B$ connected, we have that (14) holds. The base case $m = 1$ holds trivially.

Let $m \in {\mathbb {N}}$ and suppose that for any recurrent graph *G* on which the potential kernel is well defined and for any subset $B \subset v(G)$ with $|B| = m$ and $v(G) \setminus B$ connected we have that (14) and (15) are satisfied for each $x \in B$.

In this case,

$$\begin{aligned} q_B(w) = \lim _{n\rightarrow \infty } {\mathbb {P}}_{w}(T_{A_n} < T_B){\mathcal {R}}_{\text {eff} }(B \leftrightarrow A_n) \end{aligned}$$

by assumption exists and does not depend on the sequence $(A_n)_{n \ge 1}$, so we also have that $a^{G_B}(\cdot , B) = q_B(\cdot )$ by (9). Remark 3.8 then shows us that $a^{G_B}(\cdot , y)$ is well defined for *any* $y \in v(G_B)$ and hence we know that the potential kernel is well defined on $G_B$ too.

**Induction.** Let *G* be a recurrent graph for which the potential kernel is well defined and let $B \subset v(G)$ be a finite set such that $|B| = m + 1$ and $v(G) \setminus B$ is connected. Fix $x \in B$. We split into two cases, depending on *x*:

(i) the removal of *x* from *G* disconnects all components of $B \setminus \{x\}$ from infinity in *G* or
(ii) it does not.

We begin with the easy case. Suppose we are in situation (i). We have that for all $w \notin B$ (for *n* large enough)

$$\begin{aligned} {\mathbb {P}}_{A_n}(T_w< T_x) = {\mathbb {P}}_{A_n}(T_w < T_{B}) \quad \text {and} \quad {\mathcal {R}}_{\text {eff} }(w \leftrightarrow B) = {\mathcal {R}}_{\text {eff} }(w \leftrightarrow x). \end{aligned}$$

The limit on the left-hand side exists as the potential kernel is well defined, see Corollary 3.3, and hence $\lim _{n \rightarrow \infty } {\mathbb {P}}_{A_n}(T_w < T_{B}) {\mathcal {R}}_{\text {eff} }(w \leftrightarrow B)$ exists and equals *a*(*w*, *x*). Moreover, we also have

$$\begin{aligned} q_B(w) = a(w, x) - {\mathbb {E}}_{w}[a(X_{T_B}, x)] = a(w, x) - a(x, x) = a(w, x) \end{aligned}$$

which proves the result for this choice of *x*.

We move on to the more interesting case (ii). Since we are not in case (i), we can find a set $B' \subset B$ with $|B'| = m$ and $v(G) {\setminus } B'$ connected (indeed, since we are not in case (i), there is at least a path going from some vertex in *B* to infinity, without touching *x*, and removing from *B* the last vertex in *B* visited by this path provides such a set $B'$). Take *y* to be the vertex such that $\{y\} = B \setminus B'$.

Since $|B'| = m$, we have by the induction hypothesis that the potential kernel $a^{G_{B'}}(\cdot , \cdot )$ is well defined. Pick $w \in v(G)$ such that $w \notin B$, which we can view also as a vertex in $G_B$ and $G_{B'}$. Fix *n* so large that both *B* and *w* are not in $A_n$. Using (9) we have that

$$\begin{aligned} a_{A_n}^{G_{B'}}(w, B') = {\mathcal {R}}_{\text {eff} }(B' \leftrightarrow A_n){\mathbb {P}}_w(T_{A_n} < T_{B'}). \end{aligned}$$

We focus on the probability appearing on the right-hand side. By the law of total probability and the strong Markov property of the simple random walk, we have

$$\begin{aligned} {\mathbb {P}}_w(T_{A_n}< T_{B'})&= {\mathbb {P}}_w(T_{y}< T_{A_n}< T_{B'}) + {\mathbb {P}}_w(T_{A_n}< T_{y} \wedge T_{B'}) \\&= {\mathbb {P}}_w(T_{y}< T_{A_n} \wedge T_{B'}){\mathbb {P}}_{y}(T_{A_n}< T_{B'}) + {\mathbb {P}}_w(T_{A_n} < T_{B'} \wedge T_{y}). \end{aligned}$$

Since *G* (and hence $G_{B'}$) is recurrent, we have that ${\mathcal {R}}_{\text {eff} }(B' \leftrightarrow A_n) \sim {\mathcal {R}}_{\text {eff} }(x \leftrightarrow A_n) \sim {\mathcal {R}}_{\text {eff} }(B \leftrightarrow A_n)$ where $a_n \sim b_n$ means $a_n / b_n \rightarrow 1$ as $n \rightarrow \infty$. Taking $n \rightarrow \infty$ in the above identity after multiplying by ${\mathcal {R}}_{\text {eff} }(B' \leftrightarrow A_n)$ and using once more recurrence, we deduce that

$$\begin{aligned} a^{G_{B'}}(w, B') = {\mathbb {P}}_w(T_{y}< T_{B'})a^{G_{B'}}(y, B') + \lim _{n\rightarrow \infty } {\mathbb {P}}_w(T_{A_n} < T_{B}) {\mathcal {R}}_{\text {eff} }(A_n \leftrightarrow B), \end{aligned}$$

because the potential kernel on $G_{B'}$ is well defined by assumption. This implies in particular that

$$\begin{aligned} \lim _{n\rightarrow \infty } {\mathbb {P}}_w(T_{A_n}< T_B) {\mathcal {R}}_{\text {eff} }(B \leftrightarrow A_n) = a^{G_{B'}}(w, B') - {\mathbb {P}}_w(T_y < T_{B'})a^{G_{B'}}(y, B') \end{aligned}$$

exists and does not depend on the sequence $A_n$ and, thus, we deduce that $a^{G_{B}}(w, B)$ is well defined and satisfies

18 $$\begin{aligned} a^{G_B}(w, B) = a^{G_{B'}}(w, B') - {\mathbb {P}}_w(X_{T_B} = y)a^{G_{B'}}(y, B'). \end{aligned}$$

We are left to prove that $q_B(w) = a^{G_B}(w, B)$. By the induction hypothesis (because $x \in B'$) we know that

$$\begin{aligned} a^{G_{B'}}(w, B') = q_{B'}(w) = a(w, x) - {\mathbb {E}}_w[a(X_{T_{B'}}, x)]. \end{aligned}$$

Using this in (18) we get

$$\begin{aligned} a^{G_B}(w, B)&= a(w, x) - {\mathbb {E}}_w[a(X_{T_{B'}}, x)] - {\mathbb {P}}_w(X_{T_B} = y) \Big ( a(y, x) - {\mathbb {E}}_{y}[a(X_{T_{B'}}, x)]\Big ) \\&= a(w, x) - {\mathbb {P}}_{w}(X_{T_B} = y)a(y, x) - \sum _{z \in B' } {\mathbb {P}}_w(X_{T_{B'}} = z)a(z, x) \\&\quad + \sum _{z \in B'}{\mathbb {P}}_w(X_{T_B} = y) {\mathbb {P}}_{y}(X_{T_{B'}} = z) a(z, x) \\&= a(w, x) - \sum _{z \in B} {\mathbb {P}}_w(X_{T_B} = z)a(z, x), \end{aligned}$$

where in the last line we used for $z \in B'$ the equality

$$\begin{aligned} {\mathbb {P}}_w(X_{T_{B}} = z) = {\mathbb {P}}_w(X_{T_{B'}} = z) - {\mathbb {P}}_w(X_{T_{B}} = y){\mathbb {P}}_{y}(X_{T_{B'}} = z), \end{aligned}$$

which holds due to the strong Markov property for the random walk. But of course, this is the same as

$$\begin{aligned} a^{G_B}(w, B) = a(w, x) - {\mathbb {E}}_w[a(X_{T_B}, x)], \end{aligned}$$

so indeed we have that $a^{G_B}(w, B) = q_B(w)$, which finishes the induction argument. $\square$

#### Proof of (16)

Let $B \subset v(G)$ be a finite set, such that its removal does not disconnect *G*. So far, we have shown that the potential kernel is well defined on the graph $G_B$ and hence that the harmonic measure from infinity is well defined, see Corollary 3.12. In this section, we will prove (16); the third statement of Theorem 3.11. First, let us introduce some notation that will only be used here. If *G* is a graph and $B \subset v(G)$ a (finite) set, then we will write $\Delta$ for the Laplacian on *G* and $\Delta ^{G_B}$ for the Laplacian on $G_B$.

##### Proof of (16)

Let *G* be a recurrent graph on which the potential kernel is well defined, and suppose that $B \subset v(G)$ is a finite set such that $v(G) \setminus B$ is connected. Fix $x \in B$. We split into two cases:

(i) the removal of *x* disconnects $B \setminus \{x\}$ from infinity in *G* or
(ii) is does not.

In the first case, we have that ${{\text {hm}}}_{B}(x) = 1$ and also that $q_B(w) = a(w, x)$ for all $w \in B$ (indeed, for $w \notin B$ this follows immediately from (15) and for $w \in B {\setminus } \{x\}$ we have that $q_B(w) = 0 = a(w, x)$ in this case). Hence, we deduce

$$\begin{aligned} \delta _{x}(\cdot ) = \Delta (a(\cdot , x)) = \Delta (q_B(\cdot )), \end{aligned}$$

which shows the result in case (i).

In case (ii), take $B' = B \setminus \{x\}$. We will show that

19 $$\begin{aligned} \Delta _w^{G_{B'}}\big (a^{G_{B'}}(w, B') - {\mathbb {P}}_w(T_{x} < T_{B'})a^{G_{B'}}(x, B') \big ) \mid _{w = x} = {{\text {hm}}}_{B}(x), \end{aligned}$$

where $\Delta ^{G_{B'}}_u$ is the Laplacian acting on the function with variable *u*. Let us first explain how this shows the final result. As in (18) and (15) we know that (when $q_B$ is viewed as a function on $v(G_{B'})$)

$$\begin{aligned} q_B(w) = a^{G_{B'}}(w, B') - {\mathbb {P}}_w(T_{x} < T_{B'})a^{G_{B'}}(x, B'). \end{aligned}$$

Moreover, when $w \in B$, we have

$$\begin{aligned} q_B(w) = a(w, x) - {\mathbb {E}}_w[a(X_{T_B}, x)] = a(w, x) - a(w, x) = 0. \end{aligned}$$

Hence, actually,

$$\begin{aligned} (\Delta ^{G_{B'}} q_{B})(x) = \sum _{\begin{array}{c} w \sim x \\ w \in v(G_{B'}) \end{array}} q_B(w) = \sum _{\begin{array}{c} w \sim x \\ w \in v(G) \end{array}} q_B(w) = (\Delta q_B)(x), \end{aligned}$$

so that (19) implies the final result.

To prove (19), recall from (5) that^4^

$$\begin{aligned} \sum _{\begin{array}{c} u \sim x \\ u \in v(G_{B'}) \end{array}} {\mathbb {P}}_u(T_x < T_{B'}) = \frac{1}{{\mathcal {R}}_{\text {eff} }(x \leftrightarrow B')}, \end{aligned}$$

and that $\Delta ^{G_{B'}} (a^{G_{B'}}(\cdot , B')) = \delta _{B'}(\cdot )$ by Proposition 3.2. Using these two facts, we get

$$\begin{aligned}&\Delta ^{G_{B'}}_w(a^{G_{B'}}(w, x_2) - {\mathbb {P}}_w(T_{x}< T_{B'})a^{G_{B'}}(x, B'))\Big |_{w = x_1} \\&\quad = -a^{G_{B'}}(x, B')\sum _{\begin{array}{c} u \sim x \\ u \in v(G_{B'}) \end{array}} ({\mathbb {P}}_u(T_{x}< T_{B'}) - 1) \\&\quad = a^{G_{B'}}(x, B') \sum _{\begin{array}{c} u \sim x \\ u \in v(G_{B'}) \end{array}} {\mathbb {P}}_u(T_{B'} < T_{x}) \\&\quad = \frac{a^{G_{B'}}(x, B')}{{\mathcal {R}}_{\text {eff} }(x \leftrightarrow B')} = {{\text {hm}}}_{B', x}(x). \end{aligned}$$

The last equality follows from Corollary 3.3, which allows us to write

$$\begin{aligned} a^{G_{B'}}(x, B') = {{\text {hm}}}_{x, B'}(x){\mathcal {R}}_{\text {eff} }(x \leftrightarrow B'). \end{aligned}$$

This shows (16) and therefore concludes the proof of Theorem 3.11. In turn this finishes the proof that (a) is equivalent to (b) in Theorem 1.1 (see e.g. Corollary 3.12). $\square$

## Proof of Theorem 1.4

Before proceeding with the remaining equivalences we give a proof that (a) holds under the assumption of Theorem 1.4. Recall that a random graph (*G*, *o*) is **strictly subdiffusive** whenever there exits a $\beta > 2$ such that

SD $$\begin{aligned} \textbf{E}[d(o, X_n)^\beta ] \le Cn. \end{aligned}$$

We collect the following theorem of [8]. The main theorem from that paper shows that, assuming subdiffusivity, strictly sublinear harmonic functions must be constant. In fact, as already mentioned in that paper (see Example 2.10), the arguments in that paper also show that assuming *strict* subdiffusivity, even harmonic functions of at most linear growth must be constant. It is this extension which we use here, and which we quote below.

### Theorem 4.1

(Theorem 3 in [8]) Let $(G, o, X_1)$ be a strictly subdiffusive (SD), recurrent, stationary environment. A.s., every harmonic function on *G* that is of at most linear growth is constant.

We now give the proof of Theorem 1.4 using this result.

### Proof of Theorem 1.4 assuming Theorem 1.1

Let (*G*, *o*) be a unimodular graph that is almost surely strictly subdiffusive (SD) and recurrent, satisfying $\textbf{E}[\deg (o)] < \infty$. Then degree biasing (*G*, *o*) gives a reversible environment and hence, almost surely, all harmonic functions on $(G, o, X_1)$ that are at most linear are constant due to Theorem 4.1. After degree unbiasing, the same statement is true for (*G*, *o*).

We will prove that this implies that statement (a) of Theorem 1.1 holds, which (by assumption) implies (a)–(d) must be satisfied.

Let $a_1, a_2:v(G)^2 \rightarrow {\mathbb {R}}_+$ be two potential kernels arising as subsequential limits in the sense of Lemma 3.1. Fix $y \in v(G)$. By Proposition 3.2 we have that $a_i(\cdot , y)$ is of the form

$$\begin{aligned} a_i(x, y) = {\mathcal {R}}_{\text {eff} }(x \leftrightarrow y)H_i(x), \end{aligned}$$

with $0 \le H_i(x) \le 1$ for each *x* and $i = 1, 2$. Define next the map $h:v(G) \rightarrow {\mathbb {R}}$ through

$$\begin{aligned} h(x) = a_1(x, y) - a_2(x, y). \end{aligned}$$

Clearly, *h* is harmonic everywhere outside *y* by choice of the $a_i$’s and linearity of the Laplacian. Since $\Delta a_1(\cdot , y) = \Delta a_2(\cdot , y)$ by Proposition 3.2, we also get that $\Delta h(y) = 0$ and we deduce that *h* is harmonic everywhere.

Next, we notice

$$\begin{aligned} |h(x)| \le |H_1(x) - H_2(x)|{\mathcal {R}}_{\text {eff} }(y \leftrightarrow x) \le 2 d(y, x), \end{aligned}$$

implying that *h* is (at most) linear. Thus *h* must be constant. Since $h(y) = a_1(y, y) - a_2(y, y) = 0$, it follows that $h(x) = 0$ and hence we finally obtain $a_1(x, y) = a_2(x, y)$ for all $x \in v(G)$. Since $y \in v(G)$ was arbitrary, we deduce the desired result. $\square$

### Remark 4.2

Strict subdiffusivity on the UIPQ was obtained by Benajmini and Curien in the beautiful paper [6]. A result of Lee [26, Theorem 1.10] gives a more general condition which guarantees strict subdiffusivity (essentially, the graph needs to be planar with at least cubic volume growth). In particular, this applies to the UIPT.

As an example of application of Theorem 1.4 consider the Incipient Infinite percolation Cluster (IIC) of ${\mathbb {Z}}^d$ for sufficiently large *d*. By a combination of Theorem 1.2 in [24] and Theorem 1.1 in [27], one can check that the strict subdiffusivity (SD) is satisfied in all sufficiently high dimensions. The recurrence is easier to check. (Note that a weaker form of subdiffusivity can be deduced by combining [24] with [10]). In fact, it was already checked earlier that in high dimensions the backbone of the IIC is one-ended ([41]), implying also the UST is one-ended in this case.

We point out that the result should apply in dimension two (even for non-nearest neighbour walk), or for the IIC of spread-out percolation, although we do not know if strict subdiffusivity has been checked in that case.

## The sublevel set of the potential kernel

Let (*G*, *o*) be some recurrent, rooted, graph for which the potential kernel is well defined in the sense that $a_n(x, y)$ obtains a limit and this does not depend on the choice of the sequence $(A_n)_{n \ge 1}$ of finite sets of vertices going to infinity.

Fix $z \in v(G)$ and $R \in {\mathbb {R}}_+$. Recall the notation in (6) for the ball with respect to the effective resistance metric:

$$\begin{aligned} {\mathcal {B}}_{\text {eff} }(z, R) = \{x \in v(G): {\mathcal {R}}_{\text {eff} }(z \leftrightarrow x) \le R\} \end{aligned}$$

We also introduce the notation for the sublevel set of $a(\cdot , z)$ through

$$\begin{aligned} \Lambda _a(z, R) = \{x \in v(G): a(x, z) \le R\}. \end{aligned}$$

In case $z = o$, we will drop the notation for *z* and write ${\mathcal {B}}_{\text {eff} }(R)$, $\Lambda _a(R)$ for ${\mathcal {B}}_{\text {eff} }(o, R)$, $\Lambda _a(o, R)$ respectively. Although $a(\cdot , \cdot )$ fails to be a distance as it lacks to be symmetric, it *is* what we call a quasi-distance as it does satisfy the triangle inequality due to Proposition 3.6. On 2-connected graphs (where the removal of any single vertex does not disconnect the graph), we have that $a(x, y) = 0$ precisely when $x = y$. In particular, this is true for triangulations.

Let us first explain *why* we care about the sublevel sets of the potential kernel and why we will prefer it over the effective resistance balls. We will call a set $A \subset v(G)$ **simply connected** whenever it is connected (that is, for any two vertices *x*, *y* in *A*, there exists a path connecting *x* and *y*, using only vertices inside *A*) and when removing *A* from the graph does not disconnect a part of the graph from infinity. We make the following observation, which holds because $x \mapsto a(x, o)$ is harmonic outside of *o*.

### Observation

The set $\Lambda _a(R)$ is simply connected.

**Fig. 1:**
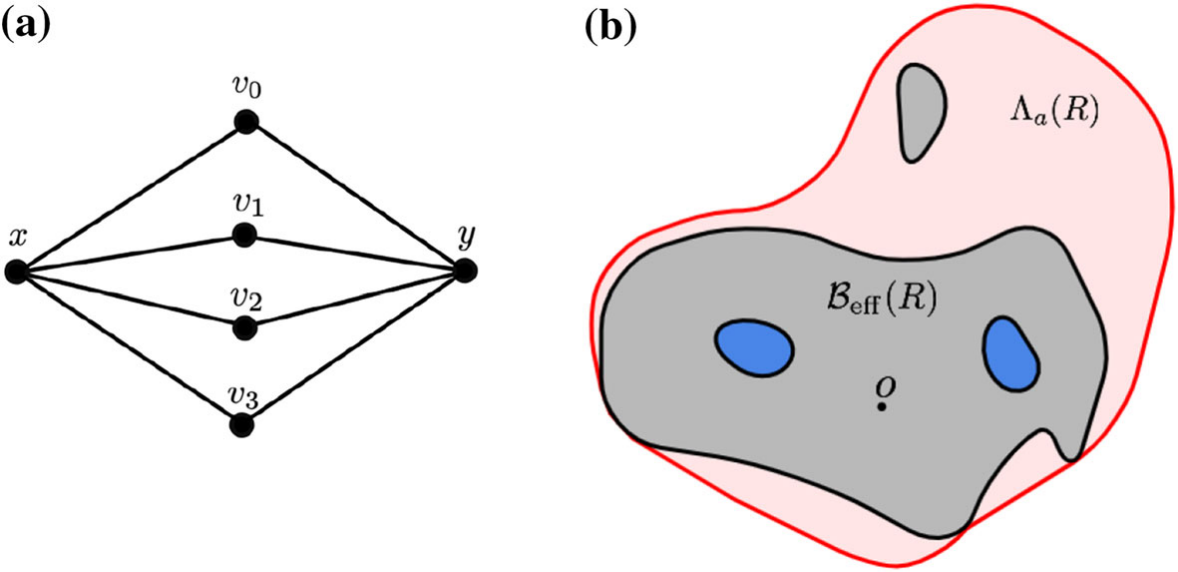
**a** Example of a graph where ${\mathcal {B}}_{\text {eff} }(z, R)$ is not connected for each *R*: the effective resistance between *x* and *y* equals 1/2, whereas the resistance between *x* and $v_i$ equals 5/8. **b** A schematic drawing. In dark gray, we see the set ${\mathcal {B}}_{\text {eff} }(R)$. The blue parts are $\overline{{\mathcal {B}}_{\text {eff} }(R)} {\setminus } {\mathcal {B}}_{\text {eff} }(R)$. The red area (and everything inside) is then the sublevel set $\Lambda _a(R)$

This is not true, in general, for ${\mathcal {B}}_{\text {eff} }(R)$. Introduce the **hull** $\overline{{\mathcal {B}}_{\text {eff} }(z, R)}$ of ${\mathcal {B}}_{\text {eff} }(z, R)$ as the set ${\mathcal {B}}_{\text {eff} }(z, R)$ together with the *finite* components of $v(G) \setminus {\mathcal {B}}_{\text {eff} }(z, R)$. Even though $\overline{{\mathcal {B}}_{\text {eff} }(z, R)}$ does not have any more “holes”, we notice that still, it is not evident (or true in general) that $\overline{{\mathcal {B}}_{\text {eff} }(z, R)}$ is *connected*. See Fig. 1a for an example.

We do notice that $\overline{{\mathcal {B}}_{\text {eff} }(z, R)} \subset \Lambda _a(z, R)$ as

$$\begin{aligned} a(x, z) = {{\text {hm}}}_{x, z}(x){\mathcal {R}}_{\text {eff} }(x \leftrightarrow z) \le {\mathcal {R}}_{\text {eff} }(x \leftrightarrow z), \end{aligned}$$

by Corollary 3.3. See also Fig. 1b for a schematic picture.

We thus get that the sets $\Lambda _a(R)$ are more regular than the sets ${\mathcal {B}}_{\text {eff} }(R)$ and if *G* is planar, they correspond to Euclidean simply connected sets.

In this section, we are interested in some properties of $\Lambda _a(R)$, that we will need to prove our Harnack inequalities. We now state the main result, which shows that $\lim _{z \rightarrow \infty } a(z, x) = \infty$, under the additional assumption that the underlying rooted graph is random and (stationary) reversible.

### Proposition 5.1

Suppose (*G*, *o*) is a reversible random graph, that is a.s. recurrent and for which the potential kernel is a.s. well defined. Almost surely, the sets $\Lambda _a(z, R)$ are finite for each $R \ge 1$ and all $z \in v(G)$, and hence $(\Lambda _a(z, R))_{R \ge 1}$ defines an exhaustion of *G*.

Although we expect this proposition to hold for all graphs where the potential kernel is well defined, we do not manage to prove the general case. In addition, the proof actually yields something slightly stronger which may not necessarily hold in full generality.

Note also that for all $R \ge 0$ we have $v(G) {\setminus } \Lambda _a(R)$ is non-empty because $x \mapsto a(x, o)$ is unbounded (to see this, assume it is bounded and use recurrence and the optional stopping theorem to deduce that *a*(*x*, *o*) would be identically zero, which is not possible since the Laplacian is nonzero at *o*). We introduce the following definition, that we will use throughout the remaining document.

### Definition 5.2

Let $$\delta \in [0, 1]$$ and $$x \in v(G)$$.

- We call *x* $(\delta , o)$-good if ${{\text {hm}}}_{o, x}(x) \ge \delta$. We will omit the notation for the root if it is clear from the context.
- We call the rooted graph (*G*, *o*) $\delta$-good if for all $\epsilon > 0$, there exist infinitely many $(\delta - \epsilon , o)$-good vertices.
- We call the rooted graph (*G*, *o*) uniformly $\delta$-good if all vertices are $(\delta , o)$-good.

Note that if the graph (*G*, *o*) is uniformly $\delta$-good for some $\delta > 0$, then actually $\Lambda _a(\delta R) \subset {\mathcal {B}}_{\text {eff} }(R)$, so that the sets $\Lambda _a(\delta R)$ are finite for each *R*. It turns out that the graph (*G*, *o*) being $\delta$-good is also enough, which is the content of Lemma 5.5 below.

Although the definition of $\delta$-goodness is given in terms of rooted graphs (*G*, *o*), the next (deterministic) lemma shows that the definition is actually invariant under the choice of the root, and hence we can can omit the root and say “*G* is $\delta$-good” instead.

### Lemma 5.3

Suppose $\delta > 0$ is such that (*G*, *o*) is $\delta$-good, then also (*G*, *z*) is $\delta$-good for each $z \in v(G)$.

### Proof

Fix $z \in v(G)$ and let $\delta > 0$ be such that (*G*, *o*) is $\delta$-good. Fix $0< \epsilon < \delta$ and denote by $G_{\alpha , o}$ the set of $(\alpha , o)$-good vertices. Take $\epsilon _1, \epsilon _2 > 0$ such that $\epsilon _1 + \epsilon _2 = \epsilon$. Then $G_{\delta - \epsilon _1, o}$ has infinitely many points by assumption.

By Corollary 3.10, we can take $R_0:= R_0(z, o, \epsilon _2)$ so large that for all $x \notin B(o, R_0)$ we have

$$\begin{aligned} |{{\text {hm}}}_{x, z}(x) - {{\text {hm}}}_{x, o}(x)| < \epsilon _2. \end{aligned}$$

This implies that any vertex $x \in G_{\delta - \epsilon _1, o} \cap B(o, R_0)^c$ must in fact be $(\delta - \epsilon , z)$-good since $\epsilon = \epsilon _1 + \epsilon _2$. This shows the desired result as $\epsilon$ was arbitrary. $\square$

The next lemma shows the somewhat interesting result that reversible environments are always $\delta$-good, with $\delta$ arbitrary close to $\frac{1}{2}$.

### Lemma 5.4

Suppose that $(G, o, X_1)$ is a recurrent reversible random rooted graph ( that is a.s. infinite) on which the potential kernel is a.s. well defined. Then a.s. (*G*, *o*) is $\frac{1}{2}$-good.

### Proof

In this proof we will write ${{\textbf {P}}}, {{\textbf {E}}}$ to denote probability respectively expectation with respect to the law of the random rooted graph (*G*, *o*). In compliance with the rest of the document, we will write ${\mathbb {P}}, {\mathbb {E}}$ to denote the probability respectively expectation w.r.t. the law of the simple random walk, conditional on (*G*, *o*).

By Lemma 5.3, we note that (*G*, *o*) being $\delta$-good is independent of the root and hence for each $\delta > 0$, the event

$$\begin{aligned} {\mathcal {A}}_{\delta } = \{(G, o) \text { is } \delta \text {-good}\} \end{aligned}$$

is invariant under re-rooting, that is

$$\begin{aligned} (G, o) \in {\mathcal {A}}_{\delta } \iff (G, x) \in {\mathcal {A}}_{\delta } \text { for all } x \in v(G). \end{aligned}$$

A natural approach to go forward would be to use that any unimodular law is a mixture of ergodic laws [2, Theorem 4.7]. We will not use this, as there is an even simpler argument in this case.

We will use the invariance under re-rooting to prove that ${\mathcal {A}}_{\delta }$ has probability one. Suppose, to the contrary, that the event ${\mathcal {A}}_{\delta }$ does not occur with probability one, so that $\textbf{P}({\mathcal {A}}_{\delta }) \in [0, 1)$. Then we can condition the law $\textbf{P}$ on ${\mathcal {A}}_{\delta }^c$ to obtain again a reversible law $\textbf{P}(\cdot \mid {\mathcal {A}}_{\delta }^c)$ (it is here that we use the invariance under re-rooting of ${\mathcal {A}}_{\delta }$, see for example [15, Exercise 15] or [2]), under which ${\mathcal {A}}_{\delta }$ has probability zero. However, we will show that $\textbf{P}({\mathcal {A}}_\delta ) > 0$ always holds when $\delta < \frac{1}{2}$, independent of what the exact underlying reversible law $\textbf{P}$ is—as long as the potential kernel is a.s. well defined and the graph is a.s. recurrent. Now, this implies that we actually need to have $\textbf{P}({\mathcal {A}}_{\delta }) = 1$, which is the desired result.

Fix $\delta < \frac{1}{2}$. We thus still need to prove that $\textbf{P}({\mathcal {A}}_\delta ) > 0$, which we do by contradiction. Assume henceforth that $\textbf{P}({\mathcal {A}}_{\delta }) = 0$. By reversibility, we get for each $n \in {\mathbb {N}}$ the equality

$$\begin{aligned} {{\textbf {E}}}[{{\text {hm}}}_{o, X_n}(X_n)] = {{\textbf {E}}}[{{\text {hm}}}_{X_n, o}(o)] = \frac{1}{2}, \end{aligned}$$

due to the fact that $(G, o, X_n)$ has the same law as $(G, X_n, o)$, which is reversibility (here, the expectation is both with respect to the environment and the walk).

As $\textbf{P}({\mathcal {A}}_{\delta }) = 0$, we can assume that a.s. there exists a (random) $N = N(G, o) \in {\mathbb {N}}$, such that for all $x \not \in B(o, N)$ we have

$$\begin{aligned} {{\text {hm}}}_{x, o}(x) \le \delta \end{aligned}$$

Also, note that the environment is a.s. null-recurrent ( as is the case for any connected, infinite recurrent graph, which follows e.g. by uniqueness of invariant of measures for recurrent graphs, Theorem 1.7.6 in [32], in conjunction with Theorem 1.7.7 of [32]). Hence we have that, (*G*, *o*)-a.s.

$$\begin{aligned} {\mathbb {P}}(X_n \text { in } B(o, N)) \rightarrow 0, \end{aligned}$$

whenever $n \rightarrow \infty$. Moreover, notice that for each *n* we have

$$\begin{aligned} {\mathbb {E}}[{{\text {hm}}}_{o, X_n}(X_n)] \le {\mathbb {P}}(X_n \text { not in } B(o, N))\delta + {\mathbb {P}}(X_n \text { in } B(o, N)). \end{aligned}$$

Since ${{\text {hm}}}_{o, X_n}(X_n) \in [0, 1]$, we can apply Fatou’s lemma (applied to *just* the expectation with respect to the law of (*G*, *o*), so that we can use the just found inequality) from which we deduce that

$$\begin{aligned} \frac{1}{2} = \limsup _{n \rightarrow \infty } \textbf{E}[{{\text {hm}}}_{o, X_n}(X_n)] \le \delta , \end{aligned}$$

which is a contradiction as $\delta < \frac{1}{2}$. $\square$

We next show that for any $\delta$-good (rooted) graph, the set $\Lambda _a(R)$ is finite for each $R \ge 1$. Combined with Lemma 5.4, this implies Proposition 5.1 in case of reversible environments. However, Lemma 5.4 shows more than just this fact. Indeed, $\Lambda _a(R)$ being finite need not imply that (*G*, *o*) is $\delta$-good for some $\delta > 0$.

### Lemma 5.5

If (*G*, *o*) is $\delta$-good for some $\delta > 0$, then $\Lambda _a(o, R)$ is finite for each $R \ge 1$.

### Proof

Let $\delta > 0$ and suppose that *G* is $\delta$-good. We will show that for each $R \ge 1$, there exists an $M \ge 1$ such that for all $x \notin B(o, M)$ we have

$$\begin{aligned} a(x, o) \ge \frac{\delta ^2 R}{8}. \end{aligned}$$

This implies the final result.

By assumption on $\delta$-goodness, for each $R \ge 1$ there exists a vertex $x_R \notin {\mathcal {B}}_{\text {eff} }(o, R)$ such that

$$\begin{aligned} {{\text {hm}}}_{x_R, o}(x_R) \ge \frac{\delta }{2} \end{aligned}$$

This implies by Corollary 3.3 that $a(x_{R}, o) \ge \frac{\delta }{2} {\mathcal {R}}_{\text {eff} }(o \leftrightarrow x_R) \ge \frac{\delta R}{2}$.

Fix $R \ge 1$ and define the set $B_R = \{o, x_{R}\}$. By Theorem 3.11, we get for all *x* the decomposition

$$\begin{aligned} a(x, o) = q_{B_R}(x) + {\mathbb {E}}_x[a(X_{T_{B_R}}, o)], \end{aligned}$$

where $q_{B_R}(\cdot )$ is the potential kernel on the graph $G_{B_R}$, which we recall is the graph *G*, with $B_R$ glued together. Since potential kernels are non-negative, we can focus our attention to the right-most term.

Take $M = M(o, x_R, \delta )$ so large that for all $x \notin B(o, M)$

$$\begin{aligned} |{{\text {hm}}}_{B_R}(x_R) - {\mathbb {P}}_x(X_{T_{B_R}} = x_R)| \le \frac{\delta }{4}, \end{aligned}$$

which is possible as the potential kernel is well defined, see Proposition 3.2 and Corollary 3.3. We deduce that for all $x \notin B(o, M)$

$$\begin{aligned} a(x, o) \ge {\mathbb {E}}_x[a(X_{T_{B_R}}, o)] \ge \frac{\delta ^2 R}{8}, \end{aligned}$$

as desired. $$\square $$

## Two Harnack inequalities

We are now ready to prove the equivalence between (c) and (a). The first part of this section deals with a classical Harnack inequality, whereas the second part of this section provides a variation thereof, where the functions might have a single pole. The first Harnack inequality (Theorem 6.4 below) does not involve Theorem 1.1.

Recall that $\Lambda _a(z, R)$ is the sublevel set $\{x \in v(G): a(x, z) \le R\}$ (for *R* not necessarily integer valued) and that *a*(*z*, *x*) defines a quasi distance on *G*. Also recall the notation ${\mathcal {B}}_{\text {eff} }(z, R) = \{x: {\mathcal {R}}_{\text {eff} }(z \leftrightarrow x) \le R\}$, for the (closed) ball with respect to the effective resistance distance.

### The standing assumptions

Throughout this section we will work with deterministic graphs *G*, which satisfy a certain number of assumptions.

#### Definition 6.1

*(Standing assumptions)* We will say that *G* satisfies the **standing assumptions** whenever it is infinite, recurrent, the potential kernel is well defined and the level sets $(\Lambda _a(z, R))_{R \ge 1}$ are finite for some (hence all by Proposition 3.6) $z \in v(G)$.

We will *not* use that (*G*, *o*) is random reversible in this section, other than to verify that is satisfies the standing Assumptions 6.1. The remainder of this section works for all (deterministic) graphs that satisfy the standing assumptions.

#### Lemma 6.2

Let (*G*, *o*) be a random unimodular graph graph with $\textbf{E}[\deg (o)] < \infty$, for which a.s. the potential kernel is uniquely defined. Then (*G*, *o*) a.s. satisfies the standing assumptions.

#### Proof

Proposition 5.1 implies that any unimodular random graph with $\textbf{E}[\deg (o)] < \infty$ that is a.s. recurrent and for which the potential kernel is a.s. well defined, the level sets $\Lambda _a(z, R)$ are finite for all *R* and $z \in v(G)$. $\square$

#### Remark 6.3

Note for instance that this implies that the UIPT/UIPQ therefore satisfies the standing assumptions, see the discussion at the end of Sect. 4. *A posteriori*, this also follows from our main Theorem (Theorem 1.1) and [1].

### Elliptic Harnack inequality

We first show that under the standing assumptions (Definition 6.1), a version of the elliptic Harnack inequality holds, where the constants are uniform over all graphs that satisfy the standing assumptions. Recall the definition of the “hull” $\overline{{\mathcal {B}}_{\text {eff} }(z, R)}$ introduced in Sect. 5.

#### Theorem 6.4

(Harnack Inequality) There exist $M, C > 1$ such that the following holds. Let *G* be a graph satisfying the Standing Assumptions 6.1. For all $z \in v(G)$, all $R \ge 1$ and all $h: \Lambda _a(z, MR) \cup \partial \Lambda _a(z, MR) \rightarrow {\mathbb {R}}_+$ that are harmonic on $\Lambda _a(z, MR)$ we have

H $$\begin{aligned} \max _{x \in \overline{{\mathcal {B}}_{\text {eff} }(z, R)}} h(x) \le C \min _{x \in \overline{{\mathcal {B}}_{\text {eff} }(z, R)}} h(x) \end{aligned}$$

#### Remark 6.5

In case the rooted graph (*G*, *o*) is in addition uniformly $\delta$-good for some $\delta$ (that is, ${{\text {hm}}}_{x, o}(x) \ge \delta$ for each *x*, see Definition 5.2), then we have that

$$\begin{aligned} \Lambda _a(\delta R) \subset {\mathcal {B}}_{\text {eff} }(R) \subset \Lambda _a(R), \end{aligned}$$

and hence the Harnack inequality above becomes a standard “elliptic Harnack inequality” for the graph equipped with the effective resistance distance. (As will be discussed below, we conjecture that many infinite models of random planar maps, including the UIPT, satisfy the property of being $\delta$-good for some nonrandom $\delta >0$.)

#### The harmonic exit measure

In the proof, we fix the root $o \in v(G)$, but it plays no special role. Define for $k \in {\mathbb {N}}$, $x \in \Lambda _a(k)$ and $b \in \partial \Lambda _a(k)$ the “harmonic exit measure”

$$\begin{aligned} \mu _k(x, b) = {\mathbb {P}}_x(X_{T_{k}} = b), \end{aligned}$$

where $T_k$ is the first hitting time of $\partial \Lambda _a(k)$. We will write

20 $$\begin{aligned} {{\textbf {G}}}_k(x, y):= {{\textbf {G}}}_{\Lambda _a(k)^c}(x, y) \end{aligned}$$

where we recall the definition of the Green function in (1). The following proposition shows that changing the starting points $x, y \in {\mathcal {B}}_{\text {eff} }(R)$, does not significantly change the exit measure $\mu _k(\cdot , b)$. The Harnack inequality will follow easily from this proposition (in fact, it is equivalent).

##### Proposition 6.6

There exist constants $\tilde{C}, M > 1$ such that for all *G* satisfying the Standing Assumptions 6.1, all $R \ge 1$ and all $x, y \in \partial \overline{{\mathcal {B}}_{\text {eff} }(R)}$ we have

$$\begin{aligned} \frac{1}{\tilde{C}}\mu _{MR}(y, b) \le \mu _{MR}(x, b) \le \tilde{C}\mu _{MR}(y, b) \end{aligned}$$

for each $$b \in \partial \Lambda _a(MR)$$.

We first prove the following lemma, giving an estimate on the number of times the simple random walk started from *x* visits *y*, before exiting the set $\Lambda _a(MR)$.

##### Lemma 6.7

For all $M_0 > 1$ and all $M \ge M_0 + 3$ there exists $C = C(M, M_0) > 1$ such that for all *G* satisfying the Standing Assumptions 6.1 and for all $R \ge 1$ we have

$$\begin{aligned} \frac{R}{C} \le \frac{{{\textbf {G}}}_{MR}(x, y)}{\deg (y)} \le CR \end{aligned}$$

for all $x \in \partial \Lambda _a(M_0R)$ and $y \in \partial \overline{{\mathcal {B}}_{\text {eff} }(R)}$.

##### Proof

Fix $M_0 > 1$ and let $M \ge M_0 + 3$. Let *G* be any graph satisfying the standing assumptions 6.1. Let $R \ge 1$, take $x \in \partial \Lambda _a(M_0R)$ and $y \in \partial \overline{{\mathcal {B}}_{\text {eff} }(R)}$. Notice that, by Lemma 3.5, we can write

21 $$\begin{aligned} \frac{{{\textbf {G}}}_{MR}(x, y)}{\deg (y)} = {\mathbb {E}}_x[a(X_{T_{MR}}, y)] - a(x, y). \end{aligned}$$

Let $z \in \Lambda _a(MR)$. Recalling that $a(\cdot , \cdot )$ is a quasi metric that satisfies the triangle inequality due to Proposition 3.6, we have, by assumption on *x* and *y* and the expression for the potential kernel in terms of harmonic measure and effective resistance (Corollary 3.3), that

22 $$\begin{aligned} a(z, y) \le a(z, o) + a(o, y) \le MR + {\mathcal {R}}_{\text {eff} }(o \leftrightarrow y) = (M + 1)R. \end{aligned}$$

Going back to (21) and upper-bounding $-a(x, y) \le 0$, we find the desired upper bound:

$$\begin{aligned} \frac{{{\textbf {G}}}_{MR}(x, y)}{\deg (y)} \le (M + 1)R. \end{aligned}$$

For the lower bound, fix again $z \in \partial \Lambda _a(MR)$. From Theorem 3.11 (and the fact that ${\mathcal {B}}_{\text {eff} }(R) \subset \Lambda _a(R)$) we obtain the equality

$$\begin{aligned} a(z, y) - {\mathbb {E}}_z[a(X_{T_{R}}, y)] = a(z, o) - {\mathbb {E}}_z[a(X_{T_{R}}, o)]. \end{aligned}$$

It follows that

23 $$\begin{aligned} a(z, y) \ge a(z, o) - {\mathbb {E}}_z[a(X_{T_R}, o)] = (M - 1)R. \end{aligned}$$

On the other hand, invoking the triangle inequality (as in (22)), we have

$$\begin{aligned} a(x, y) \le a(x, o) + a(o, y) \le (M_0 + 1)R. \end{aligned}$$

The lower-bound now follows from (21) and (23) as

$$\begin{aligned} \frac{{{\textbf {G}}}_{MR}(x, y)}{\deg (y)} \ge (M - 1)R - (M_0 + 1)R = (M - M_0 - 2)R. \end{aligned}$$

Since $M \ge M_0 + 3$, we can take $C = C(M, M_0)$ depending *only* on $M, M_0$ such that we get the result. $\square$

##### Proof of Proposition 6.6

Take $M_0 > 1, M = M(M_0)$ and $C > 1$ as in Lemma 6.7. Let *G* be a graph satisfying the standing assumptions 6.1. Fix $R \ge 1$ and let $x, y \in \partial \overline{{\mathcal {B}}_{\text {eff} }(R)}$. For $b \in \Lambda _a(MR)$ we use the last-exit decomposition (Lemma 2.1) to see

$$\begin{aligned} \mu _{MR}(x, b) = \sum _{z \in \partial \Lambda _a(M_0R)} \frac{{{\textbf {G}}}_{MR}(x, z)}{\deg (z)} \deg (z){\mathbb {P}}_z(X_{T_{MR}} = b; { T_{MR} < T_{M_0R}^+}). \end{aligned}$$

By Lemma 6.7, we have for each $z \in \partial \Lambda _a(M_0R)$

$$\begin{aligned} \frac{{{\textbf {G}}}_{MR}(z, x)}{\deg (x)} \le CR \le C^2 \frac{{{\textbf {G}}}_{MR}(z, y)}{\deg (y)}. \end{aligned}$$

We thus get, defining $\tilde{C} = C^2$, and using $\deg (\cdot )$-reversibility of the simple random walk that

$$\begin{aligned} \mu _{MR}(x, b)&\le \tilde{C} \sum _{z \in \partial \Lambda _a(M_0R)} \frac{{{\textbf {G}}}_{MR}(y, z)}{\deg (z)} \deg (z){\mathbb {P}}_z(X_{T_{MR}} = b; T_{MR} < T_{M_0R}^+)\\&= \tilde{C}\mu _{MR}(y, b), \end{aligned}$$

showing the final result. $\square$

##### Proof of Theorem 6.4

The proof of Theorem 6.4 is easy now. Indeed, let $C, M > 1$ large enough, as in Proposition 6.6 and take any graph *G* satisfying the standing assumptions and $R \ge 1$. Take $h: \Lambda _a(MR) \cup \partial \Lambda _a(MR) \rightarrow {\mathbb {R}}_+$ a function harmonic on $\Lambda _a(MR)$. Using the maximum principle for harmonic functions, we deduce that it is enough to prove

$$\begin{aligned} \max _{x \in \partial \overline{{\mathcal {B}}_{\text {eff} }(R)}} h(x) \le C \min _{x \in \partial \overline{{\mathcal {B}}_{\text {eff} }(R)}} h(x). \end{aligned}$$

Take $x, y \in \partial \overline{{\mathcal {B}}_{\text {eff} }(R)}$. By optional stopping and Proposition 6.6 we have

$$\begin{aligned} h(x) = {\mathbb {E}}_x[h(X_{T_{MR}})]&= \sum _{b \in \partial \Lambda _a(MR)} h(b) \mu _{MR}(x, b) \\&\le \tilde{C} \sum _{b \in \partial \Lambda _a(MR)} h(b) \mu _{MR}(y, b) = \tilde{C}h(y), \end{aligned}$$

showing the result. $\square$

### (a) implies (c): anchored Harnack inequality

Sometimes, one wants to apply a version of the Harnack inequality to functions that are harmonic on a big ball, but not in some vertex inside this ball (the pole). Clearly, we can only hope to compare the value of harmonic function in points that are “far away” from the pole, say on the boundary of a ball centered at the pole.

This “anchored” inequality does not always follow from the Harnack inequality as stated in Theorem 6.4. As an example, think of the graph ${\mathbb {Z}}$ with nearest neighbor connections. Pick any two positive real numbers $\alpha , \beta$ satisfying $\alpha + \beta = 1$. Then the function *h* that maps *x* to $\alpha (-x)$ when *x* is negative and to $\beta x$ when *x* is positive, is harmonic everywhere outside of 0, with $\Delta h(0) = 1$. This implies that no form of “anchored Harnack inequality” can hold.

We next present a reformulation of (a) implies (c) in Theorem 1.1. We will use it to prove results for the “conditioned random walk” as introduced in Sect. 7.

#### Theorem 6.8

(Anchored Harnack inequality) There exists a $C < \infty$ such that the following holds. Let *G* be a graph satisfying the Standing Assumptions 6.1. For $z \in v(G)$, $R \ge 1$ and all $h: v(G) \rightarrow {\mathbb {R}}_+$ that are harmonic outside of *z* and satisfy $h(z) = 0$, we have

aH $$\begin{aligned} \max _{x \in \partial \Lambda _a(z, R)} h(x) \le C \min _{x \in \partial \Lambda _a(z, R)} h(x). \end{aligned}$$

#### Remark 6.9

Actually, we will prove that for each $z \in v(G)$ and $R \ge 1$, there exists $\Psi _z(R) \ge R$ such that for all harmonic functions $h:\Lambda _a(z, \Psi _z(R)) \cup \partial \Lambda _a(z, \Psi _z(R)) \rightarrow {\mathbb {R}}_+$ that are harmonic on $\Lambda _a(z, \Psi _z(R)) {\setminus } \{z\}$ and $h(z) = 0$, we have

$$\begin{aligned} \max _{x \in \partial \Lambda _a(z, R)}h(x) \le C \min _{x \in \partial \Lambda _a(z, R)} h(x). \end{aligned}$$

As before, if the graph is uniformly $\delta$-good for some $\delta > 0$, we can actually take $\Psi _z(R) = MR$ for some $M = M(\delta )$ depending *only* on $\delta$.

#### Proof of Theorem 6.8

The proof will be somewhat similar to the proof of Theorem 6.4. Again, we will prove it for a given root vertex *o* to simplify our writing, but it will not matter which vertex we choose. For $k \in {\mathbb {N}}$, we will write again $T_k = T_{\Lambda _a(k)^c}$ for the first time the random walk exists the sublevel-set $\Lambda _a(k)$. Fix $k \in {\mathbb {N}}$ and $x \in \Lambda _a(k)$. Define, given a graph *G* satisfying the standing assumptions 6.1 and a root vertex *o* the exit measure

$$\begin{aligned} \nu _k(x, b) = {\mathbb {P}}_x(X_{T_k} = b, T_k < T_o), \end{aligned}$$

for $b \in \partial \Lambda _a(k)$. We begin by showing that, taking *x*, *y* in $\Lambda _a(R)$, the exit measures $\nu _k(x, \cdot )$ and $\nu _k(y, \cdot )$ are similar up to division by *a*(*x*, *o*), *a*(*y*, *o*) respectively, when *k* is large enough. Although it might seem at first slightly counterintuitive that that we need to divide by *a*(*x*, *o*), this actually means that the *conditional* exit measures ${\mathbb {P}}_w(X_{T_k} = b \mid T_{k} < T_o)$ for $w = x, y$ are comparable.

##### Proposition 6.10

There exists a $C < \infty$ such that for all graphs *G* satisfying the Standing Assumptions 6.1 with root *o*, for each $R \ge 1$, there exists a constant $\Psi (R) \ge R$ such that for all $x, y \in \Lambda _a(R) {\setminus } \Lambda _a(1)$ and all $b \in \partial \Lambda _a(\Psi (R))$ we have

$$\begin{aligned} \frac{\nu _{\Psi (R)}(x, b)}{a(x, o)} \le C \frac{\nu _{\Psi (R)}(y, b)}{a(y, o)}. \end{aligned}$$

In order to prove this proposition, we will first prove a few preliminary lemmas. We assume here that the underlying graphs satisfy the standing assumptions 6.1. The next result offers bounds on the probability that the random walk goes “far away” before hitting *o* in terms of the potential kernel.

##### Lemma 6.11

For each $z \in v(G) {\setminus } \Lambda _a(1)$ and all $M > a(z, o)$, we have

$$\begin{aligned} \frac{a(z, o)}{M + 1} \le {\mathbb {P}}_z(T_M < T_o) \le \frac{a(z, o)}{M}. \end{aligned}$$

##### Proof

This is a straightforward consequence of the optional stopping theorem. Indeed, since $(a(X_{n\wedge T_M \wedge T_o}, o))_{n \ge 0}$ is an a.s. bounded martingale,

$$\begin{aligned} a(z, o) = {\mathbb {E}}_z[a(T_{M} \wedge T_o, o)] \end{aligned}$$

and because $M \le a(w, o) \le M + 1$ for each $w \in \partial \Lambda _a(M)$ and $a(o, o) = 0$, we find

$$\begin{aligned} \frac{a(z, o)}{M + 1} \le {\mathbb {P}}_z(T_M < T_o) \le \frac{a(z, o)}{M}, \end{aligned}$$

which are the desired bounds. $\square$

##### Lemma 6.12

For each $R \ge 1$, there exist $M> M_0 > R$ such that for all $x \in \Lambda _a(R)$ and $z \in \Lambda _a(M_0)$,

$$\begin{aligned} \frac{1}{10} \le \frac{{{\textbf {G}}}_{B_M}(z, x)}{\deg (x)a(x, o)} \le 2, \end{aligned}$$

where $$B_M = \{o\} \cup \Lambda _a(M)^c$$.

##### Proof

Fix $R \ge 1$ and $x, y \in \Lambda _a(R) {\setminus } \Lambda _a(0)$. Take $M_0 = M_0(R)$ at least so large that for *all* $w \notin \Lambda _a(M_0)$ and all $z \in \Lambda _a(R) {\setminus } \Lambda _a(0)$ we have

24 $$\begin{aligned} \frac{1}{2} \le \frac{{\mathbb {P}}_w(T_z < T_o)}{{{\text {hm}}}_{z, o}(z)} \le 2. \end{aligned}$$

This is possible because ${\mathbb {P}}_w(T_z < T_o)$ converges to ${{\text {hm}}}_{z, o}(z)$ for all *z*, $\Lambda _a(R)$ is finite, and uniformity in *w* outside $\Lambda _a(M_0)$ follows just as in Corollary 3.9 (otherwise, we can construct a sequence $w_{M}$ of vertices going to infinity such that ${\mathbb {P}}_{w_{M}}(T_z < T_o)$ does *not* converge to ${{\text {hm}}}_{z, o}(z)$). Fix next $M = 5M_0$ and $B_M = \{o\} \cup \Lambda _a(M)^c$.

Take $z \in \Lambda _a(M_0)$. By choice of *M* and Lemma 6.11, we have

25 $$\begin{aligned} {\mathbb {P}}_z(T_M < T_o) \le \frac{M_0}{M} \le \frac{1}{5}. \end{aligned}$$

Using the strong Markov property of the walk we get

26 $$\begin{aligned} \begin{aligned}&{{\textbf {G}}}_{B_M}(z, x) = {{\textbf {G}}}_o(z, x) - {\mathbb {P}}_z(T_M< T_o)\sum _{b \in \partial \Lambda _a(M)} {\mathbb {P}}_z(X_{T_{M}} = b \mid T_{M} < T_o){{\textbf {G}}}_o(b, x). \end{aligned} \end{aligned}$$

The definition of the Green function and Corollary 3.3 allow us to write

$$\begin{aligned} \frac{{{\textbf {G}}}_o(z, x)}{\deg (x)} = {\mathbb {P}}_z(T_x < T_o) {\mathcal {R}}_{\text {eff} }(x \leftrightarrow o) \quad \text {and} \quad a(x, o) = {{\text {hm}}}_{x, o}(x){\mathcal {R}}_{\text {eff} }(x \leftrightarrow o), \end{aligned}$$

which implies that

$$\begin{aligned} \frac{{{\textbf {G}}}_{o}(z, x)}{\deg (x)a(x, o)} = \frac{{\mathbb {P}}_z(T_x< T_o)}{{{\text {hm}}}_{x, o}(x)} \quad \text {and} \quad \frac{{{\textbf {G}}}_o(b, x)}{\deg (x)a(x, o)} = \frac{{\mathbb {P}}_b(T_x < T_o)}{{{\text {hm}}}_{x, o}(x)}, \end{aligned}$$

for each $b \in \Lambda _a(M)$. Thus (26) is equivalent to

27 $$\begin{aligned} \frac{{{\textbf {G}}}_{B_M}(z, x)}{\deg (x)a(x, o)}= & {} \frac{{\mathbb {P}}_z(T_x< T_o)}{{{\text {hm}}}_{x, o}(x)} - {\mathbb {P}}_z(T_M< T_o) \nonumber \\{} & {} \quad \sum _{b \in \partial \Lambda _a(M)} {\mathbb {P}}_z(X_{T_M} = b \mid T_M< T_o) \frac{{\mathbb {P}}_b(T_x < T_o)}{{{\text {hm}}}_{o, x}(x)}. \end{aligned}$$

Hence, by (25) and using (24) twice with $w = z$ and $w = b$ respectively in (27) we get

$$\begin{aligned} \frac{1}{2} - \frac{2}{5} \le \frac{G_{o, \partial _M}(z, x)}{\deg (x)a(x, o)} \le 2, \end{aligned}$$

which is the desired result. $\square$

##### Proof of Proposition 6.10

Just as in the proof of Proposition 6.6, we use the last-exit decomposition to see

$$\begin{aligned} \frac{\nu _M(x, b)}{a(x, o)} = \sum _{z \in \partial \Lambda _a(k)} \frac{{{\textbf {G}}}_{o, \partial _M}(x, z)}{a(x, o)\deg (z)} \deg (z) {\mathbb {P}}_z(X_{T_M} = b; T_M < T_k^+). \end{aligned}$$

This implies that

$$\begin{aligned} \frac{\nu _M(x, b)}{a(x, o)} \le 20 \frac{\nu _M(y, b)}{a(y, o)}. \end{aligned}$$

We are left to define $\Psi (R) = M$ and $C = 20$ (which thus does not depend on the graph) to obtain the desired result. $\square$

Finishing the proof of Theorem 6.8 is now straightforward. Indeed, fix $C > 1$ as in Proposition 6.10. Given a (rooted) graph *G* satisfying the standing assumptions 6.1, take $\Psi$ also as in Proposition 6.10. Let $R \ge 1$ and $h: \Lambda _a(\Psi (R)) \rightarrow {\mathbb {R}}_+$ harmonic outside *o*; with $h(o) = 0$. Fix $x, y \in \Lambda _a(R)$. By optional stopping, which holds as $\Lambda _a(\Psi (R))$ is finite,

$$\begin{aligned} h(x)&= \int _{\partial \Lambda _a(\Psi (R))} h(b) \nu _{\Psi (R)}(x, b) \\&\le C\frac{a(x, o)}{a(y, o)} \int _{\partial \Lambda _a(\Psi (R))} h(b) \nu _{\Psi (R)}(y, b) = C \frac{a(x, o)}{a(y, o)}h(y). \end{aligned}$$

This shows the desired result when $x, y \in \partial \Lambda _a(R)$. $\square$

### (c) implies (a)

Let $(V_R)_R$ be any sequence of connected subsets of *v*(*G*) satisfying $o \in V_{R} \subset V_{R + 1}$, $|V_R| < \infty$ for all *R* and $\cup _{R \ge 1} V_R = v(G)$.

#### Proposition 6.13

Suppose that the (rooted) graph (*G*, *o*) satisfies the anchored Harnack inequality with respect to the sequence $(V_R)_{R \ge 1}$ and some (non-random) constant *C*: for all $h: v(G) \rightarrow {\mathbb {R}}_+$ harmonic outside possibly *o* and such that $h(o) = 0$,

$$\begin{aligned} \max _{x \in \partial V_R} h(x) \le C \min _{x \in \partial V_R} h(x). \end{aligned}$$

In this case, the potential kernel *a*(*x*, *o*) is well defined.

We take some inspiration from [36], although the strategy goes back in fact to a paper of Ancona [3]. Pick some sequence $e = (e_{R})_{R \ge 1}$ on *v*(*G*) satisfying $e_R \in \partial V_R$.

#### Lemma 6.14

Suppose *G* satisfies the anchored Harnack inequality. Let $R \ge 1$ and suppose that *h*, *g* are two positive, harmonic functions on $\Lambda _{a}(\Psi (R)) \setminus \{o\}$ vanishing at *o*. We have

$$\begin{aligned} \max _{x \in V_R \setminus \{o\}} \frac{h(x)}{g(x)} \le C^2 \frac{h(e_R)}{g(e_R)}. \end{aligned}$$

#### Proof

Fix $R \ge 1$ and let *h*, *g* be as above. Write $T_R = T_{\partial V_R}$. By optional stopping, $h(o) = 0$ and the Harnack inequality, we get

$$\begin{aligned} h(x) = {\mathbb {P}}_x(T_R< T_o){\mathbb {E}}_{x}[h(X_{T_R}) \mid T_R< T_o] \le C h(e_R) {\mathbb {P}}_x(T_R < T_o) \end{aligned}$$

for all $x \in V_R \setminus \{o\}$. Similarly, we obtain

$$\begin{aligned} g(x) \ge \frac{1}{C}g(e_R){\mathbb {P}}_x(T_R < T_o) \end{aligned}$$

for $x \in V_R \setminus \{o\}$. Combining this, we find

$$\begin{aligned} \frac{1}{C}\frac{h(x)}{h(e_R)} \le {\mathbb {P}}_x(T_R < T_o) \le C\frac{g(x)}{g(e_R)}, \end{aligned}$$

showing the final result. $\square$

#### Proof of Proposition 6.13

We follow closely Section 3.2 in [36]. We will show that whenever $h_1, h_2:v(G) \rightarrow [0, \infty )$ are harmonic functions on $v(G) {\setminus } \{o\}$, vanishing at *o*, such that $h_1(e_1) = h_2(e_1)$, we have $h_1 = h_2$. The result then follows as we can pick $h_1(\cdot )$ and $h_2(\cdot )$ to be two subsequential limits of $a_{A_n}(\cdot , o)$ (for possibly different sequences $(A_n)$ going to infinity), and rescaling so that they are equal at $e_1$.

Consider $h_1, h_2: v(G) \rightarrow [0, \infty )$ harmonic functions on $v(G) \setminus \{o\}$, vanishing at *o*. Assume without loss of generality that $h_1(e_1) = h_2(e_1) = 1$. By Lemma 6.14 we get that there is some appropriate (large) *M* which does not depend on $h_1, h_2$, for which

28 $$\begin{aligned} \frac{1}{M}\frac{h_1(x)}{h_1(e_R)} \le \frac{h_2(x)}{h_2(e_R)} \le M \frac{h_1(x)}{h_1(e_R)}, \end{aligned}$$

for all $x \in V_R$ and $R \ge 1$. It follows that (setting $x = e_1$)

$$\begin{aligned} \frac{1}{M} h_1(e_R) \le h_2(e_R) \le M h_1(e_R). \end{aligned}$$

Using this in (28) and letting $R \rightarrow \infty$, we obtain

29 $$\begin{aligned} \frac{1}{M^2} \le \frac{h_2(x)}{h_1(x)} \le M^2, \end{aligned}$$

for all $x \in v(G) \setminus \{o\}$. Define recursively, for $i \ge 3$,

30 $$\begin{aligned} h_i(x) = h_{i - 1}(x) + \frac{1}{M^2 - 1}(h_{i - 1}(x) - h_1(x)). \end{aligned}$$

It is straightforward to check that $h_i$ is non-negative (as follows from an iterated version of (29)) and harmonic outside *o*. Since *M* did not depend on $h_1, h_2$, and because $h_i(e_1) = 1$ also, we obtain that

31 $$\begin{aligned} \frac{1}{M^2} \le \frac{h_i(x)}{h_1(x)} \le M^2. \end{aligned}$$

On the other hand, it is straightforward to check that the recursion (30) can be solved explicitly to get:

$$\begin{aligned} h_i(x) = \left( \frac{M^2}{M^2 - 1} \right) ^{i - 2}(h_2(x) - h_1(x)) + h_1(x). \end{aligned}$$

Unless $h_1(x) = h_2(x)$, this grows exponentially, which is incompatible with (31). Therefore $h_1(x) = h_2(x)$. $\square$

#### Remark 6.15

The proof above makes it clear that if the potential kernel is uniquely defined (i.e. if (a) holds), then any function $h:v(G) \rightarrow {\mathbb {R}}_+$ satisfying $\Delta h(x) = 0$ for all $x \in v(G) {\setminus } \{o\}$ and for which $h(o) = 0$, is of the form $\alpha a(x, o)$ for some $\alpha \ge 0$.

#### Remark 6.16

If *G* is reversible, and satisfies the anchored Harnack inequality, then it satisfies (a) as a consequence of the above. It therefore satisfies the standing assumptions: in particular, by Theorem 6.4 holds so it also satisfies the Elliptic Harnack Inequality (EHI). We have therefore proved that anchored Harnack inequality (AHI) $\implies$ (EHI) at least for reversible random graphs, which is not a priori obvious.

## Random walk conditioned to not hit the root

Let (*G*, *o*) be a rooted graph. We will assume **throughout this section** that it satisfies the *standing assumptions* of Definition 6.1, i.e., it is recurrent, the potential kernel is well defined and the potential kernel tends to infinity. In this section, we will define what we call the conditioned random walk (CRW), which is the simple random walk on *G*, conditioned to never hit the root *o* (or any other vertex). Of course, a priori this does not make sense as the event that the simple random walk *X* will never hit *o* has probability zero. However, we can take the Doob $a(\cdot , o)$-transform and use this to define the CRW. We make this precise below.

We apply some of the results derived earlier to answer some basic questions about CRW. For example: is there a connection between the harmonic measure from infinity and the hitting probability of points (and sets)? What is the probability that the CRW will ever hit a given vertex? Do the traces of two independent random walks intersection infinitely often? Does the random walk satisfy a Harnack inequality? Does it satisfy the Liouville property? The answers will turn out to be yes for all of the above, and the majority of this section is devoted to proving such statements. These properties play a crucial role in our proof of one-endedness in the next section.

In a series of papers studying the conditioned random walk ([13, 18, 35], see also the lecture notes by Popov [34]), the following remarkable observation about the CRW $({\hat{X}}_t, t\ge 0)$ on ${\mathbb {Z}}^2$ was made. Let

$$\begin{aligned} \widehat{q}(y) = {\mathbb {P}}( \widehat{X}_t = y \text { for some }t \ge 0) = {\mathbb {P}}( \widehat{T}_y < \infty ), \end{aligned}$$

then $\lim _{y \rightarrow \infty } \widehat{q}(y) = 1/2$, even though asymptotically the conditioned walk ${\hat{X}}$ looks very similar to the unconditioned walk.

One may wonder if such a fact holds in the generality of stationary random graphs for which the potential kernel is well defined. This question was in fact an inspiration for the rest of the paper. Unfortunately, we are not able to answer this question in generality, but believe it should not be true in general. In fact, on random planar maps in the universality class of Liouville quantum gravity with parameter $\gamma \in (0,2)$ (which includes the CRT-mated maps discussed below), we expect

32 $$\begin{aligned} 0< \liminf _{y \rightarrow \infty } \widehat{q}(y)< 1/2< \limsup _{y \rightarrow \infty } \widehat{q}(y) <1, \end{aligned}$$

with every possible value in the interval between $\liminf _{y \rightarrow \infty } \widehat{q}(y)$ and $\limsup _{y \rightarrow \infty } \widehat{q}(y)$ a possible subsequential limit. See also Conjecture 9.2. We will prove the upper-bound of (32) and a form of the lower bound on CRT-mated maps in Theorem 9.1. The fact that every possibly value between $\liminf _{y \rightarrow \infty } \widehat{q}(y)$ and $\limsup _{y \rightarrow \infty } \widehat{q}(y)$ will have a subsequential limit converging to it, holds in general and will be proved in Proposition 7.7.

### Definition and first estimates

Instead of the graph distance or effective resistance distance, we will work with the quasi distance *a*(*x*, *y*). Recall the definition $\Lambda _a(y, R):= \{x \in v(G): a(x, y) \le R\}$ and $\Lambda _a(R) = \Lambda _a(o, R)$. We will fix $y = o$, but we note that in the random setting, it is of no importance that we perform our actions on the root (in that setting, everything here is conditional on some realization (*G*, *o*)).

We can thus define the **conditioned random walk** (CRW), denoted by $\widehat{X}$, as the so called Doob *h*-transform of the simple random walk, with $h(x) = a(x, o)$. To avoid unnecessarily loaded notations, we will in fact denote $a(x) = a(x,o)$ in the rest of this section.

To be precise, let *p*(*x*, *y*) denote the transition kernel of the simple random walk on *G*. Then the transition kernel of the CRW is defined as

$$\begin{aligned} \widehat{p}(x, y) = {\left\{ \begin{array}{ll} \frac{a(y)}{a(x)} p(x, y), &{} \quad x \not = 0\\ 0, &{} \quad \text {else} \end{array}\right. }. \end{aligned}$$

It is a standard exercise to show that $\widehat{p}$ indeed defines a transition kernel. To include the root *o* as a possible starting point for the CRW, we will let $\widehat{X}_1$ have the law ${\mathbb {P}}_o(\widehat{X}_1 = x) = a(x)$, and then take the law of the CRW afterwards. In this case, we can think of the CRW as the walk conditioned to never return to *o*.

We now collect some preliminary results, starting with transience, and showing that the walk conditioned to hit a far away region before returning to the origin converges to the conditioned walk, as expected.

We will write $\widehat{T}_A$ for the first hitting time of a set $A \subset v(G)$ by the conditioned random walk, and $\widehat{T}_x$ when $A = \{x\}$. We will also denote $\widehat{T}_R = \widehat{T}_{v(G) {\setminus } \Lambda _a(R)}$. We recall that $a(\cdot , \cdot )$ satisfies a triangle inequality (see Proposition 3.6) and hence we have the growth condition

33 $$\begin{aligned} a(x) \le a(y) + 1 \end{aligned}$$

for two neighboring sites *x*, *y* since $a(x, y) \le 1$ in this case.

#### Proposition 7.1

Let $x \in v(G) {\setminus } \{o\}$ and $\widehat{X}$ the CRW starting from *x*. Then

(i) The walk $\widehat{X}$ is transient.
(ii) The process $n \mapsto 1/a(\widehat{X}_{n \wedge \widehat{T}_N})$ is a martingale, where $N = \{y: y \sim o\}$

#### Proof

The proof of (ii) is straightforward since $1/ a( \widehat{X}_{n \wedge \widehat{T}_N})$ is the Radon–Nikdoym derivative of the usual simple random walk with respect to the conditioned walk. (i) then follows from the fact that $a(y) \rightarrow \infty$ along at least a sequence of vertices. Indeed, fix $2< r <R$ large and $y \in v(G) {\setminus } \Lambda _a(r + 1)$. By optional stopping (since 1/*a*(*y*) is bounded)

$$\begin{aligned} \frac{1}{a(y)} = {\mathbb {E}}_y\left[ \frac{1}{a(\widehat{X}_{\widehat{T}_R \wedge \widehat{T}_r})}\right] \ge \frac{1}{r + 1}{\mathbb {P}}_y(\widehat{T}_r < \widehat{T}_R) + \frac{1}{R + 1}{\mathbb {P}}_y(\widehat{T}_R \le \widehat{T}_r). \end{aligned}$$

Rearranging gives

34 $$\begin{aligned} {\mathbb {P}}_y(\widehat{T}_r < \widehat{T}_R) \le \frac{\frac{1}{a(y)} - \frac{1}{R + 1}}{\frac{1}{r + 1} - \frac{1}{R + 1}}. \end{aligned}$$

Taking $R \rightarrow \infty$, we see that ${\mathbb {P}}_y(\widehat{T}_r< \infty ) \le (r+1) / (a(y)) < 1$, showing that the chain is transient. $\square$

We now check (as claimed earlier) that the conditioned walk $\widehat{X}$ can be viewed as a limit of simple random walk conditioned on an appropriate event of positive (but vanishingly small) probability.

#### Lemma 7.2

Uniformly over all choices of $m\ge 1$ and paths $\varphi = (\varphi _0, \ldots , \varphi _m) \subset \Lambda _a(R)$, as $R \rightarrow \infty$,

$$\begin{aligned}{} & {} {\mathbb {P}}_{x}((X_0, \ldots , X_m) \\{} & {} \quad = (\varphi _0, \ldots , \varphi _m) \mid T_{R} < T_o^+) = {\mathbb {P}}_x( (\widehat{X}_0, \ldots , \widehat{X}_m) \\{} & {} \quad = (\varphi _0, \ldots , \varphi _m) )(1 + o(1)). \end{aligned}$$

#### Proof

The proof is similar to [34, Lemma 4.4]. Assume here that $x \not = o$ for simplicity. The proof for $x = o$ follows after splitting into first taking one step and, comparing this, and then do the remainder. Let us first assume that the end point $\varphi _m$ of $\varphi$ lies in $\partial \Lambda _a(R)$. Then

$$\begin{aligned} {\mathbb {P}}_x(({\widehat{X}}_0, \ldots , {\widehat{X}}_m) = \varphi ) = \frac{a(\varphi _m)}{a(\varphi _0)} {\mathbb {P}}_{x}((X_0, \ldots , X_m) = \varphi ). \end{aligned}$$

Since $\varphi _m \in \partial \Lambda _a(R)$, we know that $a(\varphi _m) \in (R, R + 1]$ due to (33). By optional stopping, we see

$$\begin{aligned} a(x) = {\mathbb {P}}_x(T_R< T_o) {\mathbb {E}}_x[a(X_{T_R}) \mid T_R < T_o], \end{aligned}$$

and also $a(X_{T_R}) \in (R, R+1]$. We thus find that

35 $$\begin{aligned} {\mathbb {P}}_x(T_R < T_o) = \frac{a(x)}{R}(1 + o_R(1)). \end{aligned}$$

Combining this, we get

$$\begin{aligned} {\mathbb {P}}_x((X_0, \ldots , X_m) = \varphi \mid T_R < T_o)&= \frac{{\mathbb {P}}_x((X_0, \ldots , X_m) = \varphi )}{a(x)} R(1 + o(1)). \end{aligned}$$

Now let $\varphi$ be an arbitrary path in $\Lambda _a(R)$ starting from *x*, then by the Markov property,

$$\begin{aligned}&{\mathbb {P}}_x( (X_0, \ldots , X_m) = \varphi \mid T_R< T_o) \\&\quad = {\mathbb {P}}_x ((X_0,\ldots , X_m) = \varphi ) {\mathbb {P}}_{\varphi _m} (T_R< T_o)/ {\mathbb {P}}_x( T_R< T_o) \\&\quad = {\mathbb {P}}_x ((X_0,\ldots , X_m) = \varphi ) a(\varphi _m)/ a(x) (1+ o(1))\\&\quad = {\mathbb {P}}_x ( (\widehat{X}_0, \ldots , \widehat{X}_m) = \varphi ) (1+ o(1)), \end{aligned}$$

as desired. $$\square $$

#### Remark 7.3

Note that in Lemma 7.2, the paths are allowed to depend on *R* and may have a divergent length so long as they remain in $\Lambda _a (R)$. For an arbitrary exhaustion $(G_R)_{R\ge 1}$ of *G*, we have the following slightly weaker statement: uniformly over all paths $\varphi$ of *fixed* length *m*,

$$\begin{aligned} {\mathbb {P}}_x((X_0, \ldots , X_m) = \varphi \mid T_{G_R^c} < T_o) = {\mathbb {P}}((\widehat{X}_0, \ldots , \widehat{X}_m) = \varphi )(1 + o(1)). \end{aligned}$$

Indeed, if *G* satisfies the standing assumptions then $\inf _{x: a(x, o) \ne 0} a(x) > 0$. Take $A_R = G_R^c$ which is a sequence of sets going to infinity. Then

$$\begin{aligned} \frac{a_{R}(y, o)}{a_R(x, o)} = \frac{a(y, o)}{a(x, o)}(1 + o(1)) \end{aligned}$$

holds uniformly in *y* and *x*, if $R \rightarrow \infty$. From this, the statement above follows using the last few lines of the proof of Lemma 7.2.

#### The green function

We can find an explicit expression for the Green function associated to $\widehat{X}$. To that end, we define for $x, y \in v(G) {\setminus } \{o\}$

$$\begin{aligned} \widehat{{{\textbf {G}}}}(x, y) = {\mathbb {E}}_x \left[ \sum _{n=0}^\infty \mathbbm {1}_{\widehat{X}_n = y}\right] , \end{aligned}$$

which is well defined as $\widehat{X}$ is transient (also, the well-definition would follow from the proof below, which provides yet another way to see that the CRW is transient).

##### Proposition 7.4

Let $$x, y \in v(G) \setminus \{o\}$$. Then

$$\begin{aligned} \frac{\widehat{{{\textbf {G}}}}(x, y)}{\deg (y)} = \frac{a(y, o)}{a(x, o)} \frac{{{\textbf {G}}}_o(x, y)}{\deg (y)} = \frac{a(y, o)}{a(x, o)} \big (a(x, o) - a(x, y) + a(o, y)\big ). \end{aligned}$$

##### Proof

Fix $x, y \in v(G) \setminus \{o\}$. For definiteness we take the exhaustion $\Lambda _a(R)$ of *G* here, but we need not to, any exhaustion would work. Define for $R \ge 1$ the truncated Green function:

$$\begin{aligned} \widehat{{{\textbf {G}}}}_R(x, y):= {\mathbb {E}}_x \left[ \sum _{n = 0}^{\widehat{T}_R - 1} \mathbbm {1}_{{\widehat{X}}_n = y} \right] . \end{aligned}$$

We denote $A_R = (\Lambda _a(R))^c \cup \{o\}$ and will show that

36 $$\begin{aligned} \widehat{{{\textbf {G}}}}_R(x, y) = \frac{a(y)}{a(x)} {{\textbf {G}}}_{A_R}(x, y), \end{aligned}$$

from which the result follows when *R* goes to infinity. Fix $R \ge 1$ and notice the following standard equality, which follows from the Markov property of the CRW:

$$\begin{aligned} \widehat{{{\textbf {G}}}}_R(x, y) = \frac{{\mathbb {P}}_x(\widehat{T}_y< \widehat{T}_R)}{{\mathbb {P}}_y(\widehat{T}_y^+ < \widehat{T}_R)} \end{aligned}$$

We first deal with the numerator. From the definition of the CRW we get

37 $$\begin{aligned} {\mathbb {P}}_x(\widehat{T}_y< \widehat{T}_R) = \frac{a(y)}{a(x)} {\mathbb {P}}_x(T_y < T_R \wedge T_o). \end{aligned}$$

Indeed, just sum over all paths $\varphi$ taking *x* to *y*, and which stay inside $\Lambda _a(R) \setminus \{o\}$. Then each path has as endpoint *y*, and the probability that the simple random walk will take any of these paths is nothing but ${\mathbb {P}}_x(T_y < T_R \wedge T_o)$.

We can deal with the denominator in a similar fashion, only this time we note that the beginning and end point are the same. Hence, the *a*(*y*)-terms cancel and we get

$$\begin{aligned} \widehat{{{\textbf {G}}}}_R(x, y) = \frac{a(y)}{a(x)} \frac{{\mathbb {P}}_x(T_y< T_{o} \wedge T_R)}{{\mathbb {P}}_y(T_y^+ < T_o \wedge T_R)} = \frac{a(y)}{a(x)} {{\textbf {G}}}_{A_R}(x, y). \end{aligned}$$

This shows the first equality appearing in Proposition 7.4 upon taking $R \rightarrow \infty$. The second statement follows from Proposition 3.6. $\square$

### Hitting probabilities for conditioned walk

Suppose $\widehat{X}$ and $\widehat{Y}$ are two independent CRW’s. We will begin by describing hitting probabilities of points and sets and use this to prove that the traces of $\widehat{X}$ and $\widehat{Y}$ intersect infinitely often a.s.

We begin giving a description of the hitting probability of a vertex *y* by the CRW started from *x*. Although it is a rather straightforward consequence of the expression for the Green function of the CRW, it is still remarkably clean.

#### Lemma 7.5

Let $$x, y \in v(G) \setminus \{o\}$$, then

$$\begin{aligned} {\mathbb {P}}_{x}(\widehat{T}_y< \infty ) = \frac{{{\text {hm}}}_{y, o}(y){\mathbb {P}}_y(T_x < T_o)}{{{\text {hm}}}_{x, o}(x)}. \end{aligned}$$

#### Proof

Note that for $x \not = y$ we have

$$\begin{aligned} \widehat{{{\textbf {G}}}}(x, y) = {\mathbb {P}}_x(\widehat{T}_y < \infty )\widehat{{{\textbf {G}}}}(y, y), \end{aligned}$$

so that by Proposition 7.4 and Corollary 3.3 we find

$$\begin{aligned} {\mathbb {P}}_x(\widehat{T}_y< \infty )&= \frac{\widehat{{{\textbf {G}}}}(x, y)}{\widehat{{{\textbf {G}}}}(y, y)} = \frac{a(y,o)}{a(x, o)}\frac{\frac{{{\textbf {G}}}_o(x, y)}{\deg (y)}}{a(y, o) + a(o, y)} \\&= \frac{{{\text {hm}}}_{y, o}(y) {\mathcal {R}}_{\text {eff} }(o \leftrightarrow y) \frac{{{\textbf {G}}}_o(x, y)}{\deg (y)}}{{{\text {hm}}}_{x, o}(x) {\mathcal {R}}_{\text {eff} }(o \leftrightarrow x){\mathcal {R}}_{\text {eff} }(o \leftrightarrow y)} \\&= \frac{{{\text {hm}}}_{y, o}(y) {\mathbb {P}}_y(T_x < T_o)}{{{\text {hm}}}_{x, o}(x)}, \end{aligned}$$

as desired. $$\square $$

Since the potential kernel is assumed to be well defined, we also have that ${\mathbb {P}}_y(T_x < T_o) \rightarrow {{\text {hm}}}_{o, x}(x)$ as $y \rightarrow \infty$ due to Corollary 3.3, and hence we deduce immediately the next result.

#### Corollary 7.6

Write $\widehat{q}(y) = {\mathbb {P}}_o(\widehat{T}_y < \infty )$. We have that

$$\begin{aligned} \liminf _{y \rightarrow \infty } \widehat{q}(y) = \liminf _{y \rightarrow \infty } {{\text {hm}}}_{o, y}(y) \end{aligned}$$

and the same with ‘limsup’ instead of ‘liminf’.

In particular, it is true that on *transitive* graphs that are recurrent and for which the potential kernel is well defined, by symmetry one always has $\widehat{q}(y) \rightarrow \frac{1}{2}$. This gives another proof to a result of [34] on the square lattice once it has been established that the potential kernel is uniquely defined. There are multiple ways to show the latter, including using the tools from this paper, e.g., by proving an anchored Harnack inequality as in Corollary 1.3, or by showing that sublinear harmonic functions are constant, and showing that the effective resistance grows sublinearly (in fact logarithmically).

We can now prove that the subsequential limits of the hitting probabilities $\widehat{q}(y)$ define an interval, as promised before. Note that this proposition is fairly general: it does not require the underlying graphs to be unimodular, only for the graph to satisfy the standing assumption (Definition 6.1, i.e. recurrence, existence of potential kernel and convergence to infinity of the potential kernel).

#### Proposition 7.7

Let $o \in V$ be fixed and $\widehat{q}(y) = {\mathbb {P}}_o(\widehat{T}_y < \infty )$.

For each $q \in [\liminf _{y \rightarrow \infty } \widehat{q}(y), \limsup _{y \rightarrow \infty } \widehat{q}(y)]$, there exists a sequence of vertices $(y_n)_{n \ge 1}$ going to infinity such that

$$\begin{aligned} \lim _{n\rightarrow \infty } \widehat{q} (y_n) = q. \end{aligned}$$

#### Proof

Assume that there exist $q_1 < q_2$ such that there are sequences $(y^1_n)_{n \ge 1}$ and $(y^2_n)_{n \ge 1}$ going to infinity for which $\lim _{n\rightarrow \infty } \widehat{q}(y^i_n) = q_i$, but there does not exists a sequence $y_n$ going to infinity for which $q_1< \lim _{n \rightarrow \infty } \widehat{q}(y_n) < q_2$. We will derive a contradiction. We do so via the following claim.

#### Claim

For each $\epsilon > 0$, there exists an $N = N(G, o, \epsilon )$ such that for each neighboring vertices $x, y \notin B(o, N)$, we have

$$\begin{aligned} |\widehat{q}(x) - \widehat{q}(y)| < \epsilon . \end{aligned}$$

To see this claim is true, we use Lemma 7.5 and Corollary 3.10 to get the existence of $N_1$ such that

38 $$\begin{aligned} |\widehat{q}(z) - {{\text {hm}}}_{o, z}(z)| < \frac{\epsilon }{4} \end{aligned}$$

for all $z \notin B(o, N_1)$. Next, pick $N_2$ such that all $z \notin B(o, N_2)$ have ${\mathcal {R}}_{\text {eff} }(o \leftrightarrow z) > \frac{4}{\epsilon }$. Let $x, y \notin B(o, N_1 \vee N_2)$ be neighbors. Due to (33) we have $a(x) - a(y) \le 1$ and by the triangle inequality for effective resistance also ${\mathcal {R}}_{\text {eff} }(o \leftrightarrow y) \le {\mathcal {R}}_{\text {eff} }(o \leftrightarrow x) + 1$. Hence, using the expression $a(x) = {{\text {hm}}}_{x, o}(x) {\mathcal {R}}_{\text {eff} }(o \leftrightarrow x)$ of Corollary 3.3, we deduce that

$$\begin{aligned} {{\text {hm}}}_{x, o}(x) \frac{{\mathcal {R}}_{\text {eff} }(o \leftrightarrow y) - 1}{{\mathcal {R}}_{\text {eff} }(o \leftrightarrow y)} - {{\text {hm}}}_{y, o}(y) \le \frac{a(x) - a(y)}{{\mathcal {R}}_{\text {eff} }(y \leftrightarrow o)} \le \frac{1}{{\mathcal {R}}_{\text {eff} }(o \leftrightarrow y)}, \end{aligned}$$

which implies by choice of $N_2$ that in fact

39 $$\begin{aligned} {{\text {hm}}}_{x, o}(x) - {{\text {hm}}}_{o, y}(y) < \frac{\epsilon }{2}. \end{aligned}$$

Thus, taking together equations (38) and (39) we obtain

$$\begin{aligned} \widehat{q}(x) - \widehat{q}(y) \le \epsilon . \end{aligned}$$

Since *x*, *y* are arbitrary neighbors, this implies the claim when taking $N = N_1 \vee N_2$.

By Corollary 3.13, we know that the graph *G* is one-ended as the potential kernel is assumed to be well defined. Take $\epsilon > 0$ so small that $q_2 > q_1 + 3 \epsilon$. By assumption on $q_1, q_2$, we thus have that for each *n* large enough, there exist two neighboring vertices $x, y \notin B(o, n)$ satisfying

$$\begin{aligned} \widehat{q}(y)> q_2 - \epsilon> q_1 + 2\epsilon > \widehat{q}(x) + \epsilon , \end{aligned}$$

so that $\widehat{q}(y) > \widehat{q}(x) + \epsilon$, a contradiction. $\square$

### Harnack inequality for conditioned walk

Notice that the conditioned random walk viewed as a Doob *h*-transform may be viewed as a random walk on the original graph *G* but with new conductances by

$$\begin{aligned} \widehat{c}(x, y) = a(x)a(y) \end{aligned}$$

for each edge $\{x, y\} \in e(G)$. Indeed the symmetry of this function is obvious, as is non-negativity, and since *a* is harmonic for the original graph Laplacian $\Delta$,

$$\begin{aligned} \pi (x):= \sum _{y \sim x} \widehat{c}(x, y) = \sum _{y \sim x} a(y) a(x) = { \deg (x)}a(x)^2, \end{aligned}$$

we get that the random walk associated with these conductances coincides indeed with our Doob *h*-transform description of the conditioned walk.

We can thus consider the network $(G, \widehat{c} \,)$, which is transient by Proposition 7.1. It will be useful to consider the graph Laplacian $\widehat{\Delta }$, associated with these conductances, defined by setting

$$\begin{aligned} (\widehat{\Delta } h)(x) = \sum _{y \sim x} \widehat{c}(x, y)(h(y) - h(x)). \end{aligned}$$

for a function *h* defined on the vertices of *G*, although *h* does not need to be defined at *o*. We will say that a function $h: v(G){\setminus } \{o\} \rightarrow {\mathbb {R}}$ is harmonic (w.r.t. the network $(G, \widehat{c} \,)$) whenever $\widehat{\Delta } h \equiv 0$. This is of course equivalent to

$$\begin{aligned} h(x) = {\mathbb {E}}_x[h(\widehat{X}_1)] \end{aligned}$$

for each $$x \in v(G) \setminus \{o\}$$.

It might be of little surprise that the anchored Harnack inequality (Theorem 6.8) implies (in fact, it is equivalent but this will not be needed) to an elliptic Harnack inequality on the graph *G* with conductance function $\widehat{c}$, at least when viewed from the root (i.e., for exhaustion sequences centered on the root *o*).

#### Proposition 7.8

There exists a $C > 1$ such that the following holds. Suppose the graph *G* satisfies the standing assumptions. Let ${\hat{h}}: v(G) {\setminus } \{o\} \rightarrow {\mathbb {R}}_+$ be harmonic with respect to $(G, \widehat{c} \,)$. Then for each $R \ge 1$,

$$\begin{aligned} \max _{x \in \partial \Lambda _a(R)} {\hat{h}}(x) \le C \min _{x \in \partial \Lambda _a(R)} {\hat{h}}(x). \end{aligned}$$

Alternatively, the max and the min could be taken over $\Lambda _a(R)$ instead of $\partial \Lambda _a(R)$.

#### Proof

Since the graph follows the standing assumptions it satisfies the anchored Harnack inequality of Theorem 6.8. Furthermore, ${\hat{h}}(x)$ is $\widehat{\Delta }$-harmonic if and only if

$$\begin{aligned} h(x) = {\left\{ \begin{array}{ll} a(x) {\hat{h}}(x) &{} \quad \text { if } x \ne o\\ 0 &{} \quad \text { if } x = o \end{array}\right. } \end{aligned}$$

is harmonic for $\Delta$ away from *o*. Thus we can apply Theorem 6.8 to it at $z =o$. Since also $|a(x) - R | \le 1$ for $x \in \partial \Lambda _a(R)$, this anchored Harnack inequality implies the anchored Harnack inequality for ${\hat{h}}$ immediately. To obtain the corresponding inequality where the extrema are taken on $\Lambda _a(R)$, we use the maximum principle (see Section 2.1 in [30]) with respect to the $\widehat{c}$ conductances; note that these extrema may not be attained at *o*. $\square$

As a corollary we obtain the Liouville property for $\widehat{X}\;{:}\; (G, \widehat{c})$ does not carry any non-constant, bounded harmonic functions. This implies in turn that the invariant $\sigma$-algebra $\mathcal {I}$ of the CRW is trivial.

#### Corollary 7.9

The network $(G, \widehat{c} \,)$ satisfies the Liouville property, that is: any function $h:v(G) \setminus \{o\} \rightarrow {\mathbb {R}}$ that is harmonic and bounded must be constant.

#### Proof

Let *h* be a bounded, harmonic function with respect to $(G, \widehat{c} \,)$. Define the function

$$\begin{aligned} \hat{h} = h - \inf _{x \in v(G)} h(x), \end{aligned}$$

which is non-negative and harmonic. Moreover, for each $\epsilon > 0$, there exists an $x_{\epsilon }$ such that $\hat{h}(x_{\epsilon }) \le \epsilon$. Take $R_{\epsilon }$ so large that $x_{\epsilon } \in \Lambda _a(R_{\epsilon })$. By the Harnack inequality (Proposition 7.8) we deduce that for all $x \in \Lambda _a(R_{\epsilon })$,

$$\begin{aligned} 0 \le \hat{h}(x) \le C\hat{h}(x_{\epsilon }) \le C\epsilon . \end{aligned}$$

Since $\epsilon$ is arbitrary, and *C* does not depend on $R_{\epsilon }$ nor $\epsilon$, this shows the desired result. $\square$

### Recurrence of sets

We will say that a set $A \subset v(G)$ is recurrent for the chain $\widehat{X}$ whenever there exist $x \in v(G)$ such that

$$\begin{aligned} {\mathbb {P}}_x(\widehat{X}_n \in A \text { i.o.}) = 1, \end{aligned}$$

where $\text { i.o.}$ is short-hand for ‘infinitely often’. Since $(G, \widehat{c} \,)$ satisfies the Liouville property, such probabilities are 0 or 1, hence the definition of *A* being recurrent is independent of the choice of *x*. If a set is not recurrent, it is called transient. Since $\widehat{X}$ is transient, any finite set *A* is transient too. Notice, by the way, that the definition above is equivalent to saying that *A* is recurrent whenever ${\mathbb {P}}_x(\widehat{T}_A < \infty ) = 1$ for all $x \in v(G)$.

We capture next some results, relating recurrence and transience of sets to the harmonic measure from infinity. Recall Definition 5.2 of $\delta$-good points: *x* is $\delta$-good whenever ${{\text {hm}}}_{x, o}(x) \ge \delta$.

#### Lemma 7.10

If *A* has infinitely many $\delta$-good points for some $\delta > 0$, then *A* is recurrent for $\widehat{X}$.

#### Proof

This follows from a Borel–Cantelli argument. Indeed, fix $$x \in v(G)$$. Let $$\delta $$ be as in the assumption. Take $$(g_{i})_{i = 1}^\infty $$ a sequence of $$\delta $$-good points in *A*, with $$a(g_i) >i $$ (which we can clearly find as $$\Lambda _a(i)$$ is finite whereas *A* has infinitely many good points).

We will define two sequences $(R_i)_{i \ge 1}$ and $(M_i)_{i \ge 1}$. Set $M_0 = 0$ and $R_0 = 0$. Suppose we have defined $R_i, M_{i - 1}$ already. Set $a_i = a(g_{R_i})$ and $\Lambda _i = \Lambda _a (a_i)$, and note that by definition $g_{R_i} \in \Lambda _i$. Take $M_i$ so large (and greater than $R_i$) that

40 $$\begin{aligned} {\mathbb {P}}_z(\widehat{X} \text { ever hits } \Lambda _i) \le \frac{\delta }{4}, \text { uniformly over } z \in \Lambda _a (M_i)^c \end{aligned}$$

This is possible since $\Lambda _i$ is finite and ${\hat{X}}$ is transient by Proposition 7.1 and more precisely the hitting probabilities of a finite set converge to zero (see (34)). Next, let $R_{i + 1}$ be so large (and greater than $M_i$) that

41 $$\begin{aligned} \frac{{\mathbb {P}}_y(T_x < T_o)}{ {{\text {hm}}}_{o, x}(x)} \ge 1/2, \end{aligned}$$

for $y = g_{R_{i+1}}$ and all $x \in \Lambda _a(M_i)$. This is possible because $\Lambda _a(M_i)$ is finite and hitting probabilities converge to harmonic measure from infinity, by Corollary 3.3. We can also require without loss of generality that $g_{R_{i+1}} \in \Lambda _a(M_i)^c$.

Suppose that $$x \in \Lambda _a (M_{i-1})$$ is arbitrary. We first claim that from *x* it is reasonably likely that the conditioned walk $$\widehat{X}$$ will hit $$ y = g_{R_{i}}$$. Indeed, note that by Lemma 7.5, and since *y* is $$\delta $$-good and (41) holds,

$$\begin{aligned} {\mathbb {P}}_x( \widehat{T}_{y}< \infty )&= {{\text {hm}}}_{y,o} (y) \frac{{\mathbb {P}}_y (T_x< T_o)}{{{\text {hm}}}_{x,o} (x)} \ge \delta /2 \end{aligned}$$

On the other hand, conditionally on hitting $y = g_{R_i}$, the conditioned walk $\widehat{X}$ is very likely to do so before exiting $\Lambda _{a} (M_i)$ (let us call $\tau _i$ this time). Indeed, by the strong Markov property at $\tau _i$ and (40),

$$\begin{aligned} {\mathbb {P}}_x ( \widehat{T}_y > \tau _i, \widehat{T}_y < \infty ) \le \sup _{z \in \Lambda _a(M_i)^c} {\mathbb {P}}_z ( \widehat{X} \text { ever hits } \Lambda _i) \le \delta /4. \end{aligned}$$

Therefore,

$$\begin{aligned} {\mathbb {P}}_x (\widehat{T}_y < \tau _i) \ge \frac{\delta }{2} - \frac{\delta }{4} = \frac{\delta }{4}. \end{aligned}$$

Let $E_i$ be the above event, i.e., $E_i = \{ \widehat{T}_{g_{R_i}} < \tau _i\}$. Since $x \in \Lambda _a(M_{i-1})$ in the above lower bound is arbitrary, it follows from the strong Markov property at time $\tau _{i-1}$ that ${\mathbb {P}}(E_i | {\mathcal {F}}_{\tau _{i-1}} ) \ge \delta /4$, where $({\mathcal {F}}_n)_{n \ge 0}$ is the filtration of the conditional walk. By Borel–Cantelli we conclude immediately that $E_i$ occurs infinitely often a.s. (for the conditioned walk), which concludes the proof. $\square$

### Infinite intersection of two conditioned walks

We finish this section by showing that two independent conditioned random walks have traces that intersect infinitely often (for simplicity here the CRW’s are conditioned to not hit the same root *o*). We manage to prove this under two (different) additional assumptions. We start by adding the assumption that (*G*, *o*) is random and reversible.

#### Proposition 7.11

Suppose that (*G*, *o*) is a reversible random graph, such that a.s. it is recurrent and a.s. the potential kernel is well defined. Let $\widehat{X}$, $\widehat{Y}$ be two independent CRW’s started from $x, y \in v(G)$ respectively, avoiding *o*. Then a.s.

$$\begin{aligned} {\mathbb {P}}(|\{\widehat{X}_n: n \in {\mathbb {N}}\} \cap \{\widehat{Y}_n: n \in {\mathbb {N}}\} | = \infty ) =1. \end{aligned}$$

#### Proof

Suppose that (*G*, *o*) has infinitely many $\frac{1}{3}$-good vertices, and call the set of such vertices $A:= A(G, o)$. Since there are various sources of randomness here, it is useful to recall that ${\mathbb {P}}$ the underlying probability measure ${\mathbb {P}}$ is always conditional on the rooted graph (*G*, *o*). Then by Lemma 7.10, we know that

$$\begin{aligned} {\mathbb {P}}(|\{\widehat{X}_n: n \in {\mathbb {N}}\} \cap A| = \infty ) = 1. \end{aligned}$$

Now, consider the set $B = \{\widehat{X}_n: n \in {\mathbb {N}}\} \cap A$. By definition, every point in *B* is $\frac{1}{3}$-good. Since $\widehat{Y}$ is independent of $\widehat{X}$ (when conditioned on (*G*, *o*)), we can use Lemma 7.10 again to see that on an event of ${\mathbb {P}}$-probability 1,

$$\begin{aligned} {\mathbb {P}}(|\{\widehat{Y}_n: n \in {\mathbb {N}}\} \cap B| = \infty \mid \widehat{X}) = 1 \end{aligned}$$

Taking expectation w.r.t. $\widehat{X}$ we deduce that the traces of $\widehat{X}$ and $\widehat{Y}$ intersect infinitely often ${\mathbb {P}}$-almost surely, conditioned on (*G*, *o*) having infinitely many $\frac{1}{3}$-good vertices. However, Lemma 5.4 implies that, under our assumptions on (*G*, *o*), this happens with $\textbf{P}$-probability one, showing the desired result. $\square$

A consequence of the infinite intersection property is that the (random) network $(G, \widehat{c} \,)$ is a.s. Liouville. Therefore we get a new proof of the already obtained (in Corollary 7.9) Liouville property for the conditioned walk, but this time without using the Harnack inequality. On the other hand, [7] proved that for planar graphs, the Liouville property is in fact equivalent to the infinite intersection property and this results extends without any additional arguments to the case of planar networks.

By Proposition 7.8 and Corollary  7.9 we thus also obtain as a corollary of [7] the infinite intersection property for planar networks such that the potential kernel tends to infinity.

#### Proposition 7.12

Suppose *G* is a (not necessarily random reversible) planar graph satisfying the standing assumptions. Let $\widehat{X}$ and $\widehat{Y}$ be two independent CRW’s avoiding *o*, started from $x, y \in v(G)$ respectively. Then

$$\begin{aligned} {\mathbb {P}}(|\{\widehat{X}_n: n \in {\mathbb {N}}\} \cap \{\widehat{Y}_n: n \in {\mathbb {N}}\}| = \infty ) = 1. \end{aligned}$$

#### Remark 7.13

It will be useful for us to recall that the infinite intersection property implies that one walk intersects the loop-erasure of the other:

$$\begin{aligned} {\mathbb {P}}(|\{{{\text {LE}}}(\widehat{X})_n: n \in {\mathbb {N}}\} \cap \{\widehat{Y}_n: n \in {\mathbb {N}}\}| = \infty ) = 1, \end{aligned}$$

where ${{\text {LE}}}(\widehat{X})$ is the Loop Erasure of $\widehat{X}$ and $\widehat{X}, \widehat{Y}$ are two independent CRW’s that don’t hit the root *o*, started from *x*, *y* respectively. See [31] for this result.

## (a) implies (d): one-endedness of the uniform spanning tree

In this section we show that the uniform spanning tree is one ended, provided that the underlying graph is unimodular. In particular, we prove that (a) implies (d) in Theorem 1.1.

### Theorem 8.1

Suppose that (*G*, *o*) is a reversible, recurrent graph for which the potential kernel is a.s. well defined and such that $a(x) \rightarrow \infty$ along any sequence $x \rightarrow \infty$. Then the uniform spanning tree is one-ended almost surely.

Before proving this theorem, we start with a few preparatory lemmas. We will write $\mathcal {T}$ to denote the uniform spanning tree and begin by recalling the following “path reversal” for the simple random walk, a standard result. In what follows, fix the vertex $o \in v(G)$, but it plays no particular role other than to simplify the notation.

### Lemma 8.2

(Path reversal) Let $o, u \in v(G)$. For any subset of paths $\mathcal {P}$

$$\begin{aligned} {\mathbb {P}}_u((X_n: n \le T_o) \in \mathcal {P} \mid T_o< T_u^+) = {\mathbb {P}}_o((X_n: n \le T_u) \in \mathcal {P}' \mid T_u < T_o^+), \end{aligned}$$

where a path $\varphi \in \mathcal {P}'$ if and only if the reversal of the path is in $\mathcal {P}$.

See Exercise (2.1d) in [30]. The next result says that the random walk started from *o* and stopped when hitting *u*, conditioned to hit *u* before returning to *o* looks locally like a conditioned random walk when *u* is far away. This is an extension of Lemma 7.2 and its proof is similar.

### Lemma 8.3

For each $M \in {\mathbb {N}}$ and $\epsilon > 0$, there exists an *L* such that for all $u \notin \Lambda _a(L)$ and uniformly over all paths $\varphi$ going from *o* to $\partial \Lambda _a(M)$,

$$\begin{aligned} {\mathbb {P}}_o((X_0, \ldots , X_{T_M}) = \varphi \mid T_u < T_o^+) = {\mathbb {P}}_o((\widehat{X}_0, \ldots , \widehat{X}_{T_M}) = \varphi ) \pm \epsilon . \end{aligned}$$

### Proof

Fix $M \in {\mathbb {N}}$ and $\epsilon > 0$. Let $\varphi$ be some path *o* to $\Lambda _a(M)$ not returning to *o*. Denote by $\varphi _{end} \in \partial \Lambda _a(M)$ the endpoint of such a path. By the Markov property for the simple walk

$$\begin{aligned} {\mathbb {P}}_o((X_o, \ldots , X_{T_M}) = \varphi , T_u< T_o^+)&= {\mathbb {P}}_o((X_{o}, \ldots , X_{T_M}) = \varphi ){\mathbb {P}}_{\varphi _{end}}(T_u < T_o). \end{aligned}$$

Now, take *L* so large that uniformly over $x \in \Lambda _a(M)$ with $x \ne o$,

$$\begin{aligned} \frac{{\mathbb {P}}_{x}(T_u< T_o)}{\deg (o){\mathbb {P}}_o(T_u < T_o^+)} = a(x) \pm \epsilon \end{aligned}$$

By definition, we have that

$$\begin{aligned} {\mathbb {P}}_o(X_1 = \varphi _1) = \frac{1}{\deg (o)}, \end{aligned}$$

yet ${\mathbb {P}}_o(\widehat{X}_1 = \varphi _1) = a(\varphi _1)$. Therefore, and by definition of the *h*-transform,

$$\begin{aligned} {\mathbb {P}}_o((X_o, \ldots , X_{T_M}) = \varphi , T_u< T_o^+) = {\mathbb {P}}_o((\widehat{X}_o, \ldots , \widehat{X}_{T_M}))\frac{1}{\deg (o)a(\varphi _{end})}{\mathbb {P}}_{\varphi _{end}}(T_u < T_o), \end{aligned}$$

so that after dividing both sides through ${\mathbb {P}}_o(T_u < T_o^+)$, we have

$$\begin{aligned} {\mathbb {P}}_o((X_o, \ldots , X_{T_M}) = \varphi \mid T_u < T_o^+) = {\mathbb {P}}_o((\widehat{X}_o, \ldots , \widehat{X}_{T_M}) = \varphi ) \pm \epsilon \end{aligned}$$

as desired. $$\square $$

We will say that the graph satisfies an infinite intersection property for the CRW whenever

cIP $$\begin{aligned} {\mathbb {P}}(|\{\widehat{X}_n: n \in {\mathbb {N}}\} \cap \{{{\text {LE}}}(\widehat{Y})_n: n \in {\mathbb {N}}\}| = \infty ) = 1 \end{aligned}$$

where $\widehat{X}$ and $\widehat{Y}$ are independent.

Next, under the assumption (cIP) it holds that as $u \rightarrow \infty$, a simple random walk started at *u* is very unlikely to hit ${{\text {LE}}}(\widehat{Y})$ in *o*. This is the key property which gives one-endedness of the UST.

### Lemma 8.4

Suppose (cIP) holds, then

$$\begin{aligned} \limsup _{M \rightarrow \infty } \sup _{u \notin \Lambda _a(M)} {\mathbb {P}}_u(X_{T_{{{\text {LE}}}(\widehat{Y})}} = o) = 0, \end{aligned}$$

where *X* is a simple random walk started at *u* and $\widehat{Y}$ an independent conditioned walk started at *o*.

### Proof

Let *A* be any simple path from *o* to infinity in *G*. Then

42 $$\begin{aligned} {\mathbb {P}}_u(X_{T_{A}} = o) \le {\mathbb {P}}_u(\{X_n: n \le T_o\} \cap A = \{o\} \mid T_o < T_u^+). \end{aligned}$$

To see this, it is useful to recall that the successive excursions (or loops) from *u* to *u* forms a sequence $(Z_1, Z_2, \ldots )$ of i.i.d. paths (with a.s. finite length). Let *N* be the index of the first excursion which touches *o*. Then the law of $Z_N$, up to its hitting time of *o*, is that of ${\mathbb {P}}_u( \cdot | T_o < T_u^+)$. Furthermore, on the event $\{ X_{T_{A}} = o\}$ it is necessarily the case that:

- $$Z_1, \ldots , Z_{N-1}$$ avoid *A*.
- $Z_N$ touches *A* for the first time in *o*.

When we ignore the first point above, we therefore obtain the upper-bound (42).

By Lemma 8.2, the right hand side is equal to

$$\begin{aligned} {\mathbb {P}}_o(\{X_n: n \le T_u\} \cap A = \{o\} \mid T_u < T_o^+). \end{aligned}$$

Therefore, it suffices to show that this converges uniformly to zero over $u \in \Lambda _a(M)^c$, as $M \rightarrow \infty$.

Let $\widehat{X}$ be a CRW, started at *o*. Fix $\epsilon > 0$ and let *M* be some integer to be fixed later. Take $L = L(M, \epsilon )$ large enough so that

43 $$\begin{aligned} {\mathbb {P}}_o(\{X_n: n \le T_M\} \cap A = \{o\} \mid T_u < T_o^+) \le {\mathbb {P}}_o(\{\widehat{X}_n: n \le T_M\} \cap A = \{o\}) + \frac{\epsilon }{2},\nonumber \\ \end{aligned}$$

for all $u \notin \Lambda _a(L)$, which is possible by Lemma 8.3 (note that *L* depends only on $\epsilon$ and *M*, in particular does not depend on the choice of *A*). Next, take *M* so large that

44 $$\begin{aligned} {\mathbb {P}}(\{\widehat{X}_n: n \le T_M\} \cap \{{{\text {LE}}}(\widehat{Y})_n: n \in {\mathbb {N}}\} \ne \{o\}) \ge 1 - \frac{\epsilon }{2}, \end{aligned}$$

where $\widehat{Y}$ is an independent CRW. This is possible by the intersection property (cIP) as the expression in (44) is increasing in *M*. Hence for $u \notin \Lambda _a(L)$, combining (43) and (44), conditioning on ${{\text {LE}}}(\widehat{Y})$,

$$\begin{aligned} {\mathbb {P}}_u(X_{T_{{{\text {LE}}}(\widehat{Y})}} = o) \le \epsilon \end{aligned}$$

As $\epsilon$ was arbitrary, this shows the result. $\square$

### Wilson’s algorithm rooted at infinity

Recall Wilson’s algorithm for recurrent graphs: let $I = (v_0, v_1, \ldots )$ be any enumeration of the vertices *v*(*G*). Fix $E_0 = \{v_0\}$ and define inductively $E_{i + 1}$ given $E_i$, to be $E_i$ together with the loop erasure of an (independent) simple random walk started at $v_{i + 1}$ and stopped when hitting $E_{i}$. Set $E = E(I) = \cup _{i \ge 0} E_i$. Then Wilson’s algorithm tells us that the spanning tree *E* is in fact a uniform spanning tree (i.e., its law is the weak limit of uniform spanning trees on exhaustions) and in particular, its law does not depend on *I*, see Wilson [43] for finite graphs and e.g. [30] for infinite recurrent graphs.

Since the conditioned random walk is well defined, we can also start differently: namely take again some enumeration $I = (v_0, \ldots )$ of *v*(*G*). Define $F_0 = {{\text {LE}}}(\widehat{X})$, started at $v_0$ say and let $F_{i + 1}$ be $F_i$ together with the loop erasure of a simple random walk started at $v_{i + 1}$ and stopped when hitting $F_{i}$. Define $F = F(I) = \cup _{i \ge 0} F_i$. It is not hard to see that again, *F* is a spanning tree of *G* (the idea is that the loops formed by the walk coming back to the origin are erased anyway, so one might as well consider the conditioned walk). This is called “Wilson’s algorithm rooted at infinity”. A similar idea was first introduced for transient graphs in [11] and later defined for ${\mathbb {Z}}^2$.

#### Lemma 8.5

(Wilson’s algorithm rooted at infinity) The spanning tree *F* is a uniform spanning tree.

#### Proof

Begin with *o* and let $(z_n)_{n \ge 0}$ be some sequence of vertices going to infinity in *G*. Apply Wilson’s algorithm with the orderings $I_n:= (o, z_n, v_2, \ldots ) \equiv v(G)$, then the law of the first branch $E_1$ equals ${{\text {LE}}}(X^{z_n \rightarrow o})$ by construction, where $X^{z_n \rightarrow o}$ is (the trace of) a random walk started at $z_n$ and stopped when hitting *o*. This law converges to ${{\text {LE}}}(\widehat{X})$ as $i \rightarrow \infty$ due to first the path-reversal (Lemma 8.2) and them Lemma 8.3. Since Wilson’s algorithm is independent of the ordering of *v*(*G*), the result follows. $\square$

**Orienting the UST.** When the UST is one-ended, it is always possible to unambiguously assign a consistent orientation to the edges (from each vertex there is a unique forward edge) such that the edges are oriented towards the unique end of the tree. Although we do not of course know *a priori* that the UST is one-ended, it will be important for us to show that the tree inherits such a consistent orientation from Wilson’s algorithm rooted at infinity. Furthermore, we need to show this orientation does not depend on the ordering used in the algorithm. To see this, consider an exhaustion $G_n$ of the graph. Perform Wilson’s algorithm (with initial boundary given by the boundary of $G_n$) and some given sequence of vertices. When adding the branch containing the vertex *x* to the tree by performing a loop-erased walk starting from *x*, orient these edges uniquely from *x* to the boundary.

We point out that it is not entirely clear *a priori* that this orientation converges, or that the limit of the orientation does not depend on the exhaustion (indeed on ${\mathbb {Z}}$ the oriented tree converges but the orientation depends on the exhaustion, though the UST itself doesn’t), nor is it immediately clear that the law of the oriented tree doesn’t depend on the sequence of vertices. But this follows readily from the fact that the loops at *x* from a random walk starting from *x* are all erased, so that the branch containing *x* is obtained by loop-erasing a random walk conditioned to hit the boundary before returning to *x*, a process which has a limit as $n \rightarrow \infty$, is transient, and does not depend on the exhaustion used when we assume that the potential kernel is well defined. Thus the law of this oriented tree, call it $\mathbf {{\mathcal {T}}}_n$, has a limit $\mathbf {{\mathcal {T}}}$ as $n \rightarrow \infty$. Obviously, $\mathbf {{\mathcal {T}}}$ can be described directly in the infinite graph by adding to the construction of Lemma 8.5 the orientation we get from Wilson’s algorithm. When seen like this, it might not be immediately clear that the law of $\mathbf {{\mathcal {T}}}$ doesn’t depend on the ordering of vertices for Wilson’s algorithm. To see this, observe that the orientation of $\mathbf {{\mathcal {T}}}_n$ is identical to the one where all edges of ${\mathcal {T}}_n$ are oriented towards $\partial G_n$, and the law of ${\mathcal {T}}_n$ itself does not depend on the ordering, as discussed before. Hence $\mathbf {{\mathcal {T}}}_n$ does not depend on the ordering of vertices, and taking limits, neither does $\mathbf {{\mathcal {T}}}$.

Note that if *x*, *y* are two vertices on a bi-infinite path of $\mathbf {{\mathcal {T}}}$, then it makes sense to ask if *y* is in the past of *x* or vice-versa: exactly one of these alternatives must hold.

We are now ready to start with the proof of Theorem 8.1.

#### Proof of Theorem 8.1

Notice that if *G* is a graph satisfying the standing assumptions (Definition 6.1) and is moreover planar or random and unimodular then (almost surely), *G* satisfies the intersection property for CRW (cIP) due to Propositions 7.12 and 7.11 respectively.

Suppose (*G*, *o*) is reversible, and satisfies the standing assumptions a.s. For a vertex *x* of *G*, consider the event $\mathcal {A}_2(x)$ that there are two *disjoint and simple* paths from *x* to infinity in the UST $\mathcal {T}$, in other words there is a bi-infinite path going through *x*. Note that it is sufficient to prove

$$\begin{aligned} {\mathbb {P}}(\mathcal {A}_2(x)) = 0 \end{aligned}$$

for each $x \in v(G)$ a.s., where we remind the reader that here ${\mathbb {P}}$ is conditional given the graph (i.e., it is an average over the spanning tree ${\mathcal {T}}$). Indeed, for the tree $\mathcal {T}$ to be more than one-ended, there must at least be some simple path in $\mathcal {T}$ which goes to infinity in both directions. By biaising and unbiaising by the degree of the root to get a unimodular graph, it is sufficient to prove that ${\mathbb {P}}({\mathcal {A}}_2(o)) = 0$ a.s. Therefore it is sufficient to prove ${\textbf{P}} ({\mathcal {A}}_2(o)) = 0$, where we remind the reader that $\textbf{P}$ is averaged also over the graph. We first outline the rough idea before giving the details. Suppose for contradiction that $\textbf{P}(A_2(o)) \ge \epsilon > 0$. If this is the case then it is possible for both ${\mathcal {A}}_2(o)$ and ${\mathcal {A}}_2(x)$ to hold simultaneously, for many other vertices – including vertices far away from *o*. However, ${\mathcal {T}}$ is connected (since *G* is recurrent) and by Theorem 6.2 and Proposition 7.1 in [2], ${\mathcal {T}}$ is at most two-ended. Therefore the bi-infinite paths going through *x* and *o* must coincide: essentially, the bi-infinite path containing *o* must be almost space-filling.

Suppose *x* is in the past of *o* (which we can assume without loss of generality by reversibility). Using Wilson’s algorithm rooted at infinity to sample first the path from *o* and then that from *x*, the event ${\mathcal {A}}_2(o) \cap {\mathcal {A}}_2(x)$ requires a very unlikely behaviour: namely, a random walk starting from *x* must hit the loop-erasure of the conditioned walk starting from *o* exactly at *o*. This is precisely what Lemma 8.4 shows is unlikely, because of the infinite intersection properties.

Let us now give the details. Given *G*, we sample *k* independent random walks $(X^1, \ldots , X^k)$ from *o*, independently of ${\mathcal {T}}$, where $k = k(\epsilon )$ will be chosen below. Observe that by stationarity of (*G*, *o*), we have for every $n\ge 0$,

$$\begin{aligned} {\textbf{P}} ({\mathcal {A}}_2(X^i_n)) = {\textbf{P}} ({\mathcal {A}}_2(o)) \ge \epsilon . \end{aligned}$$

First we show that we can choose *k* such that for every *n*, there is *i* and *j* such that ${\mathcal {A}}_2(X^i_n) \cap {\mathcal {A}}_2(X^j_n)$ holds with ${\textbf{P}}$- probability at least $\epsilon /2$. Indeed fix $n\ge 0$ arbitrarily for now, write $E_i = {\mathcal {A}}_2(X^i_n)$. Then by the Bonferroni inequalities,

$$\begin{aligned} {\textbf{P}} ( \bigcup _{i=1}^k E_i) \ge \sum _{i=1}^k {\textbf{P}}(E_i) - \sum _{1\le i \ne j\le k} {\textbf{P}} (E_i \cap E_j) \end{aligned}$$

so that

$$\begin{aligned} \sum _{1\le i \ne j\le k} {\textbf{P}} (E_i \cap E_j) \ge k \epsilon - \textbf{P} ( \bigcup _{i=1}^k E_i) \ge k \epsilon - 1. \end{aligned}$$

Choose $k = \lceil 2/\epsilon \rceil$, then we deduce that for some $1\le i< j \le k$,

$$\begin{aligned} {\textbf{P}} ( E_i \cap E_j) \ge {k \atopwithdelims ()2}^{-1}. \end{aligned}$$

By stationarity (rerooting at the endpoint of the *i*th walk), and the Markov property of the walk, this implies

45 $$\begin{aligned} {\textbf{P}} ( {\mathcal {A}}_2(o) \cap {\mathcal {A}}_2 (X_{2n}) ) \ge {k \atopwithdelims ()2}^{-1}. \end{aligned}$$

When ${\mathcal {A}}_2(o) \cap {\mathcal {A}}_2 (X_{2n})$ occurs, both *o* and $X_{2n}$ are on some bi-infinite path, the two paths must coincide. By symmetry (i.e., reversibility) and invariance of the oriented tree $\mathbf {{\mathcal {T}}}$ with respect to the ordering of vertices,

46 $$\begin{aligned} {\textbf{P}} ( {\mathcal {A}}_2(o) \cap {\mathcal {A}}_2 (X_{2n}); X_{2n} \in {\textbf {Past}}(o)) \ge \delta := (1/2) {k \atopwithdelims ()2}^{-1}. \end{aligned}$$

Let $\widehat{Y}$ denote a conditioned walk starting from *o* and let ${{\text {LE}}}( \widehat{Y})$ denote its loop-erasure, and let *Z* be a random walk starting from a different vertex *x*. Now, pick *M* large enough that for any $x \in \Lambda _a(M)^c$

47 $$\begin{aligned} {\mathbb {P}}_x(Z_{T_{{{\text {LE}}}(\widehat{Y})}} = o) \le \delta /3, \end{aligned}$$

which we may by Lemma 8.4. Even though *M* is random (depending only on the graph), observe that as $n \rightarrow \infty$,

$$\begin{aligned} {\mathbb {P}}( X_{2n} \in \Lambda _a(M)) \rightarrow 1 \end{aligned}$$

since *G* is a.s. null recurrent (as is any recurrent infinite graph). Therefore by dominated convergence,

$$\begin{aligned} {\textbf{P}} (X_{2n} \in \Lambda _a(M)) \rightarrow 1. \end{aligned}$$

It follows using (46) that we may choose *n* large enough that

48 $$\begin{aligned} {\textbf{P}} ( {\mathcal {A}}_2(o) \cap {\mathcal {A}}_2 (X_{2n}) \cap \{ X_{2n } \in {\textbf {Past}}(o)\} \cap \{ X_{2n} \notin \Lambda _a(M)\}) \ge 2 \delta /3. \end{aligned}$$

To conclude, we pick *n* as above, and use Wilson’s algorithm rooted at infinity (Lemma 8.5) by first sampling the path from *o* (which is nothing else by ${{\text {LE}}}(\widehat{Y})$ and then sampling the path in $\mathbf {{\mathcal {T}}}$ from $x = X_{2n}$, by loop-erasing a random walk *Z* from this point, stopped at the time *T* where it hits ${{\text {LE}}}({\hat{Y}})$. As mentioned above, When ${\mathcal {A}}_2(x)$ and ${\mathcal {A}}_2(o)$ occur and *x* is in the past of *o*, since ${\mathcal {T}}$ is at most two-ended (by [2]), it must be that $Z_T = o$. (If we do not specify that $x \in {\textbf {Past}} (o)$ there might otherwise also be the possibility that *x* itself was directly on the loop-erasure of the conditioned walk). Hence, using (48) and (47),

$$\begin{aligned} 2\delta /3&\le {\textbf{P}} ( {\mathcal {A}}_2(o) \cap {\mathcal {A}}_2 (X_{2n}) \cap \{X_{2n} \in {\textbf {Past}} (o) \} \cap \{ X_{2n} \notin \Lambda _a(M)\}) \\&\le {\textbf{E}} (1_{\{Z_T = o\}} 1_{\{X_{2n} \notin \Lambda _a(M)\}} ) \\&\le {\textbf{E}} ( {\mathbb {P}}_{X_{2n}} (Z_T = o) 1_{ X_{2n} \notin \Lambda _a(M)} ) \le \delta /3, \end{aligned}$$

after conditioning on $X_{2n}$. This is a contradiction, and concludes the proof of Theorem 8.1 (and hence also that (a) implies (d) in Theorem 1.1). $\square$

Furthermore, (d) is already known by [11, Theorem 14.2] to imply (b), which we have already shown is equivalent to (a). This finishes the proof of Theorem 1.1.

## Harmonic measure from infinity on mated-CRT maps

Let $\textbf{P}$ denote the law of the whole plane mated-CRT map $G = G^{1}$ with parameter $\gamma \in (0,2)$ and with root *o*. We will not give a precise definition of these maps here and instead refer the reader for instance to [17] or [9]. Since $\textbf{E}[\deg (o)] < \infty$, the potential kernel is well defined (either because it is planar, or because it is strictly subdiffusive). We now discuss a more quantitative statement concerning the harmonic measure from infinity which underlines substantial differences with the usual square lattice.

We will write $B_{\text {euc} }(x, n)$ for the ball of vertices $z \in v(G)$ such that the Euclidean distance between *z* and *x* (w.r.t. the natural embedding) is at most *n*.

### Theorem 9.1

There exists a $\delta = \delta (\gamma ) > 0$ such that the following holds. Almost surely, there exits an $N \ge 1$ such that for all $x \notin B_{\text {euc} }(N)$ we have that

$$\begin{aligned} {{\text {hm}}}_{o, x}(x) \le 1 - \delta . \end{aligned}$$

In particular,

$$\begin{aligned} \textbf{P}\left( \frac{1}{2} \le \limsup _{y \rightarrow \infty } \widehat{q}(y) \le 1 - \delta \right) = 1. \end{aligned}$$

In fact, we expect the following stronger result to hold:

### Conjecture 9.2

For some (nonrandom) $a,b>0$, almost surely

49 $$\begin{aligned} a = \liminf _{y \rightarrow \infty } \widehat{q}(y) \le \limsup _{y \rightarrow \infty } \widehat{q}(y) =1 - b. \end{aligned}$$

In fact, sharp values for *a*, *b* can be conjectured by considering the minimal and maximal exponents for the LQG volume of a Euclidean ball of radius $\varepsilon$ in a $\gamma$-quantum cone, which all decay polynomially as $\varepsilon \rightarrow 0$ (see Lemma A.1 in [9]). We also conjecture that this holds for other random planar maps in the universality class of Liouville quantum gravity with parameter $\gamma \in (0,2)$, such as the UIPT.

### Remark 9.3

It is interesting to ask what happens for general unimodular random planar maps for which the potential kernel is uniquely defined, but here the conjecture does not hold in general. Take for example the *canopy tree*, i.e., the local limit in the Benjamini–Schramm sense of a sequence of binary trees of height *n*, with uniformly chosen root vertex. The canopy tree can be described as semi-infinite spine, from which finite trees hang at each vertex. Note that it is unimodular as a Benjamini–Schramm limit, and has a uniquely defined harmonic measure from infinity. (This can for instance be deduced from Theorem 1.1 since the graph is a tree, so the uniform spanning tree coincides with the canopy tree itself, which is clearly one-ended, or can be seen directly in a number of ways.) However, if we fix a vertex *x* not on the spine, and let *y* be on the spine, removing *y* disconnects *x* from infinity, so ${{\text {hm}}}_{x,y}(x) = 0$. This shows that Conjecture 9.2 cannot hold for this example.

Based on this we conjecture that $\max (a,b) < 1/2$. This would show a stark contrast with the square lattice ${\mathbb {Z}}^2$ where we recall that $a = b = 1/2$ (see e.g. [34]). The upper bound in (49) is of course stated in Theorem 9.1 so that the lower bound in (49) is what we are asking about. While we are not able to prove this, we may use the unimodularity of the law $\textbf{P}$ is unimodular, to prove a slightly weaker lower bound:

### Corollary 9.4

Let $\delta > 0$ as in the previous theorem. Then, almost surely, the asymptotic fraction of $\delta$-good points equals one or in other words, a.s.,

$$\begin{aligned} \liminf _{n \rightarrow \infty } \frac{1}{|B(n)|}|\{x \in B(n): {{\text {hm}}}_{o, x}(x) < \delta \}| = 0. \end{aligned}$$

### Proof

Let $\tilde{\textbf{P}}$ denote the law $\textbf{P}$ after degree biasing. We write $({\tilde{G}}, {\tilde{o}})$ for the random graph with law $\tilde{\textbf{P}}$.

On the one hand, by reversibility of $\tilde{\textbf{P}}$, we know that

$$\begin{aligned} \tilde{\textbf{P}}({{\text {hm}}}_{\tilde{o}, X_n}(X_n)> 1 - \delta ) = \tilde{\textbf{P}}({{\text {hm}}}_{\tilde{o}, X_n}(o) > 1 - \delta ) = \tilde{\textbf{P}}({{\text {hm}}}_{\tilde{o}, X_n}(X_n) < \delta ). \end{aligned}$$

On the other hand, by Theorem 9.1 and the reversed Fatou’s lemma, we have

$$\begin{aligned} \limsup _{n \rightarrow \infty }\tilde{\textbf{P}}({{\text {hm}}}_{\tilde{o}, X_n}(X_n) > 1 - \delta ) = 0, \end{aligned}$$

thus

$$\begin{aligned} \lim _{n \rightarrow \infty }\tilde{\textbf{P}}({{\text {hm}}}_{\tilde{o}, X_n}(X_n) < \delta ) = 0. \end{aligned}$$

The result now follows by contradiction: indeed, suppose that with positive probability, there is a positive asymptotic fraction of vertices $x \in B(n)$ which have ${{\text {hm}}}_{\tilde{o}, x}(x) < \delta$, then the random walk will spend a positive fraction of time in these points, giving a contradiction. $\square$

### Preliminaries and known results

We collect some known results about mated-CRT maps which are needed for the proof of Theorem 9.1.

#### Lemma 9.5

There exist $C = C(\gamma ) < \infty$ and $\alpha = \alpha (\gamma ) > 0$, such that for all $n \in {\mathbb {N}}$,

$$\begin{aligned} \textbf{P}\left( \frac{1}{C} \log (n) \le {\mathcal {R}}_{\text {eff} }(o \leftrightarrow \partial B_{\text {euc} }(o, n)) \le C \log (n) \right) \ge 1 - \frac{1}{\log (n)^\alpha }. \end{aligned}$$

#### Proof

This is Proposition 3.1 in [16]. $\square$

#### Lemma 9.6

There exists a $C = C(\gamma ) < \infty$ and $\alpha = \alpha (\gamma ) > 0$ such that with $\textbf{P}$-probability at least $1 - n^{-\alpha }$, for all $x \in B_{\text {euc} }(3n)$ and all $s \in [1/3, 1]$

$$\begin{aligned} \max _{z \in \partial B_{\text {euc} }(x, sn)} h(z) \le C \min _{x \in B_{\text {euc} }(x, sn)} h(z) \end{aligned}$$

whenever $h: B_{\text {euc} }(x, 3n) \cup \partial B_{\text {euc} }(x, 3n) \rightarrow {\mathbb {R}}_+$ is harmonic outside of possibly *x* and $\partial B_{\text {euc} }(x, 3n)$.

#### Proof

This is the content of Proposition 3.8 [9]. $\square$

#### Lemma 9.7

There exist $C = C(\gamma ) < \infty$ and $\alpha = \alpha (\gamma )$ such that with $\textbf{P}$-probability at least $1 - n^{-\alpha }$, for all $x \in B_{\text {euc} }(3n)$,

$$\begin{aligned} {\mathcal {R}}_{\text {eff} }(x \leftrightarrow \partial B_{\text {euc} }(x, n)) \ge \frac{1}{C} \log (n). \end{aligned}$$

#### Proof

This follows from Lemma 4.2 in [9]. $\square$

#### Proposition 9.8

Let (*G*, *o*) have the law of the mated-CRT map with parameter $\gamma$. There exist constants $C = C(\gamma )$ and $\alpha = \alpha (\gamma ) > 0$ such that

$$\begin{aligned} \textbf{P}\Big (\frac{1}{C} \log (n) \le a(x, o) \le C \log (n) \text { for all } x \in B_{\text {euc} }(o, 2n) \setminus B_{\text {euc} }(o, n) \Big ) \ge 1 - \frac{1}{\log (n)^\alpha }. \end{aligned}$$

#### Proof of Proposition 9.8

By Lemma 3.5 we know that for each $n \in {\mathbb {N}}$

$$\begin{aligned} {\mathcal {R}}_{\text {eff} }(o \leftrightarrow \partial B_{\text {euc} }(o, 2n)) = {\mathbb {E}}_o[a(X_{T_{2n}}, o)] \end{aligned}$$

(where we recall that ${\mathbb {E}}_o$ is the expectation solely on the random walk). Now, fix *n* and let $\mathcal {E}_n$ be the intersection of both events in Lemmas 9.5 and 9.6, which are properties of the graph only. Note that ${\mathcal {E}}_n$ holds with high probability over the mated-CRT maps (possibly by suitably changing the values of the constants).

Then, as $x \mapsto a(x, o)$ is harmonic outside *o*, conditional on $\mathcal {E}_{2n}$, we know that whenever $x \in B_{\text {euc} }(o, 2n) {\setminus } B_{\text {euc} }(o, n)$,

$$\begin{aligned} \frac{1}{C} a(X_{T_{2n}}, o) \le a(x, o) \le C a(X_{T_{2n}}, o), \end{aligned}$$

so that taking (random walk) expectations,

$$\begin{aligned} \frac{1}{C^2}\log (n) \le a(x, o) \le C^2\log (n). \end{aligned}$$

This is the desired result. $\square$

### Proof of Theorem 9.1

Take throughout the proof the constants $C, \alpha$ such that Lemmas 9.7, 9.6 and 9.5 and Proposition 9.8 hold simultaneously with the same constants.

#### Proof

The second statement follows immediately from the first statement, from the identity

$$\begin{aligned} \limsup _{y \rightarrow \infty } \widehat{q}(y) = \limsup _{y \rightarrow \infty } {{\text {hm}}}_{y, o}(y), \end{aligned}$$

in Corollary 7.6, and from the fact that for each $\epsilon > 0$, there are infinitely many $(\frac{1}{2}-\epsilon )$-good vertices by Lemma 5.4. We are thus left to prove the first statement.

To that end, fix $N_0$ so large that for all $n \ge N_0$,

$$\begin{aligned} \frac{n^{2/\alpha }}{(n - 1)^{2/\alpha }} \le 3. \end{aligned}$$

Define next for $m \ge 1$ the event

50 $$\begin{aligned} E_m \text { the event that } a(x, o) \le C\log (m) \text { for all } x \in B_{\text {euc} }(m). \end{aligned}$$

By Proposition 9.8, we know that $\textbf{P}(E_m^c) \le \log (m)^{-\alpha }$ and therefore,

$$\begin{aligned} \sum _{n = 1}^\infty \textbf{P}(E_{e^{n^{2/ \alpha }}}^c) < \infty . \end{aligned}$$

By Borel–Cantelli, this implies that there is some (random) $N_1 = N_1(G, o) < \infty$ such that $E_{e^{n^{2 / \alpha }}}$ occurs for all $n \ge N_1$. Suppose without loss of generality that $N_1 \ge N_0$ almost surely. In this case, it follows that

51 $$\begin{aligned} a(x, o) \le C \log |x| \qquad \text {for all} \quad x \notin B_{\text {euc} }(o, N_1). \end{aligned}$$

Next, define the events

$$\begin{aligned} \begin{gathered} H_m \text { the event that for all } x \in B_{\text {euc} }(3m) \setminus B_{\text {euc} }(m) \text { and for all } \\ h:v(G) \rightarrow {\mathbb {R}}_+ \text { harmonic outside of } x,\\ \max _{z \in \partial B_{\text {euc} }(x, |x|)} h(z) \le C \min _{z \in \partial B_{\text {euc} }(x, |x|)} h(z) \end{gathered} \end{aligned}$$

and

$$\begin{aligned} R_m \text { the event that for all } x \in B_{\text {euc} }(3m), {\mathcal {R}}_{\text {eff} }(x \leftrightarrow \partial B_{\text {euc} }(x, m)) \ge \frac{1}{C} \log (m). \end{aligned}$$

By Lemmas 9.6 and 9.7 respectively, it holds that $\textbf{P}(H_m^c) \le m^{-\alpha }$ and $\textbf{P}(R_m) \le m^{-\alpha }$. Therefore, using again a Borel–Cantelli argument, there exists some (random) $N_2 \ge N_1 \ge N_0$ such that almost surely, for all $n \ge N_2$ the events $H_{n^{2/\alpha }}$ and $R_{n^{2/\alpha }}$ occur. In particular, we know that almost surely,

52 $$\begin{aligned} {\mathcal {R}}_{\text {eff} }(x \leftrightarrow \partial B_{\text {euc} }(x, |x|)) \ge \frac{1}{C} \log |x| \qquad \text {for all} \quad x \notin B_{\text {euc} }(o, N_2) \end{aligned}$$

and almost surely

53 $$\begin{aligned} \begin{gathered} \text {For all } x \notin B_{\text {euc} }(o, N_2), \text { for all } h: v(G) \rightarrow {\mathbb {R}}_+ \text { harmonic outside of } x \\ \max _{z \in \partial B_{\text {euc} }(x, |x|)} h(z) \le C \min _{z \in \partial B_{\text {euc} }(x, |x|)} h(z). \end{gathered} \end{aligned}$$

Take $x \notin B_{\text {euc} }(o, N_2)$. Assume without loss of generality that ${{\text {hm}}}_{o, x}(x) \le \frac{1}{2}$, as otherwise we are done. Then

54 $$\begin{aligned} {\mathcal {R}}_{\text {eff} }(o \leftrightarrow x) \le 2{{\text {hm}}}_{o, x}(x){\mathcal {R}}_{\text {eff} }(o \leftrightarrow x) = 2a(x, o) \le 2C\log |x|, \end{aligned}$$

where we used Corollary 3.3 in the equality and (51) in the last inequality.

Furthermore, as $z \mapsto a(z, x)$ is harmonic outside of *x*, applying (53) first and then (52) gives

$$\begin{aligned} a(o, x) \ge \frac{1}{C} {\mathbb {E}}_x[a(X_{T_{B_{\text {euc} }(x, |x|)}}, x)] = \frac{1}{C} {\mathcal {R}}_{\text {eff} }(x \leftrightarrow \partial B_{\text {euc} }(x, |x|)) \ge \frac{1}{C^2} \log |x|. \end{aligned}$$

Combining the last equation with (54), we find

$$\begin{aligned} {{\text {hm}}}_{o, x}(o) = \frac{a(o, x)}{{\mathcal {R}}_{\text {eff} }(o \leftrightarrow x)} \ge \frac{1}{2C^3}, \end{aligned}$$

which shows the final result. $\square$

## Data Availability

The mansucript has no associated data.

## Footnotes

1 We thank Tom Hutchcroft for this wonderful analogy.

2 A note of warning: we use the combinatorial convention of normalisation for the discrete Laplacian, instead of the probabilistic one.

3 Although the proof here relies on the assumption that the limit of $a_{A_n}$ does not depend on the choice $A_n$, we note for future reference that it also applied if we replace *a* by any subsequential limit of $a_{A_n}$. See also Remark 3.8.

4 Of course, to be precise we would need to calculate the probabilities and effective resistances on the graph $G_{B'}$, but since this makes no difference in the current setting, we skip the extra notation.
